# The Promise of Exosomes in Cardiac Repair: When ‘Best Things Come in Small Packages’

**DOI:** 10.1111/jcmm.70918

**Published:** 2025-12-11

**Authors:** Anthony Yazbeck, Zena Wehbe, Yara Menassa, Alaa Abdelhamid, Assaad A. Eid, Amirhossein Sahebkar, Ali H. Eid

**Affiliations:** ^1^ Faculty of Medicine American University of Beirut Beirut Lebanon; ^2^ Vascular Biology Department, Institute of Clinical and Molecular Sciences St. George's University of London London UK; ^3^ College of Medicine, QU Health Qatar University Doha Qatar; ^4^ Biotechnology Research Center, Pharmaceutical Technology Institute Mashhad University of Medical Sciences Mashhad Iran; ^5^ Applied Biomedical Research Center Mashhad University of Medical Sciences Mashhad Iran; ^6^ Department of Basic Medical Sciences, College of Medicine, QU Health Qatar University Doha Qatar

**Keywords:** cardiovascular disease, myocardial infarction, myocardial ischaemia, regenerative medicine, stem cells

## Abstract

Myocardial ischaemia continues to be a predominant global cause of mortality, leaving survivors with compromised quality of life and significant loss of functional cardiomyocytes. The main therapeutic approach, which attempts to restore heart function, generally involves myocardial reperfusion. However, this intervention is frequently complicated by the occurrence of myocardial reperfusion injury, which undermines its therapeutic benefits. Consequently, there is an increasing focus on alternative regenerative approaches, such as stem and progenitor cell therapies. Since their initial and successful use in oncology, stem cells have emerged as promising tools for mitigating various pathological conditions. Nonetheless, their efficacy in post‐ischaemic myocardial environments is questioned due to their rapid degradation following delivery. Interestingly, small extracellular vesicles, particularly exosomes secreted by stem cells, demonstrate reparative properties akin to those of the stem cells themselves. Indeed, evidence strongly shows that exosomes derived from mesenchymal stem cells, cardiac progenitor cells, and induced pluripotent stem cells exert anti‐apoptotic and pro‐angiogenic effects in post‐ischaemic cardiomyocytes while concomitantly offering protection against myocardial reperfusion injury. In this review, we critically appraise the pivotal findings supporting the potential clinical application of stem cell‐derived exosomes, and underscore key considerations necessary to optimise their therapeutic efficacy.

## 1 Introduction

Ischaemic heart diseases remain the leading pathological cause of death and morbidity worldwide. Patients who survive acute myocardial infarction (MI) are often left with a reduced number of functional cardiomyocytes, resulting in diminished quality of life and subsequent mortality [1, 2]. Myocardial ischaemia is primarily the result of insufficient delivery of oxygen‐rich blood to the myocardium, usually owing to molecular changes within small blood vessels related to the process of aging, and from atherosclerotic plaque buildup [3, 4]. The latter develops over time and culminates in narrower and more rigid arteries such as the coronary arteries, leading to diminished blood flow to the heart [5, 6, 7, 8]. This ischaemia can also arise concomitantly with chronic diseases such as hypertension, diabetes, and inflammation [9, 10]. Ultimately, the inadequate blood supply leads to subsequent cardiomyocyte loss via apoptosis, necrosis, or autophagy. Left untreated, hypertrophy of cardiomyocytes ensues, resulting in ventricular remodelling and heart failure [11].

The main treatment of myocardial ischaemia involves restoring blood flow through percutaneous coronary intervention (PCI), an approach that is not without adverse complications. One major concern is myocardial ischaemia–reperfusion injury, a paradoxical response where the return of oxygenated blood causes oxidative stress and additional damage to the myocardium [1, 11, 12]. This clinical reality has driven the search for more effective, less invasive, and safer alternatives. One such avenue that has garnered considerable attention is stem cell therapy (SCT) [13, 14], which aims to restore damaged heart tissue by introducing cells capable of renewal, repair, and regeneration [15, 16]. Regenerative SCT involves the delivery of SCs that can produce new tissue. Treating patients with SCs following an acute MI ameliorated their left ventricular systolic function and appears to be a potential reparative modality [17].

**FIGURE 1:**
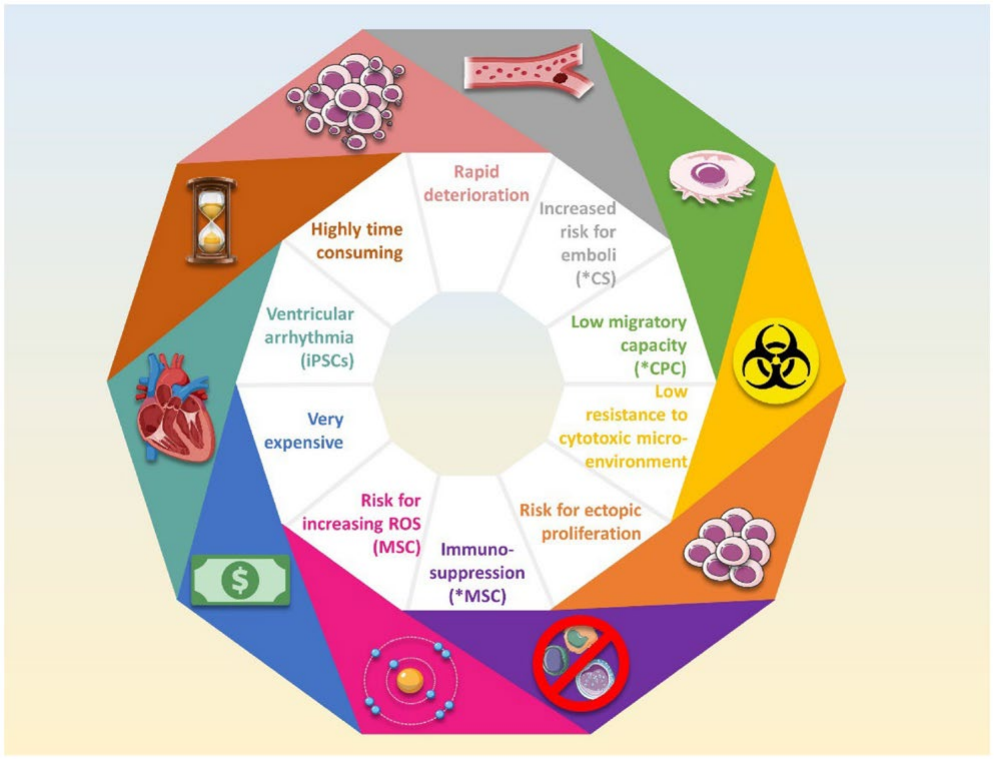
Limitations of stem cells for the treatment of myocardial infarction. Regardless of their origin, most stem cells and stem‐like cells, including cardiac stem cells (CSCs)*, cardiac progenitor cells (CPCs)*, cardiospheres (CSs), mesenchymal stem cells (MSCs)* and induced pluripotent stem cells (iPSCs)* rapidly deteriorate upon injection into myocardial tissue. Other limitations include the risk for uncontrolled proliferation and transformation into malignant cells, the possibility of inducing embolisms (CSs), as well as an immunosuppressive environment (MSCs), among others.

**FIGURE 2:**
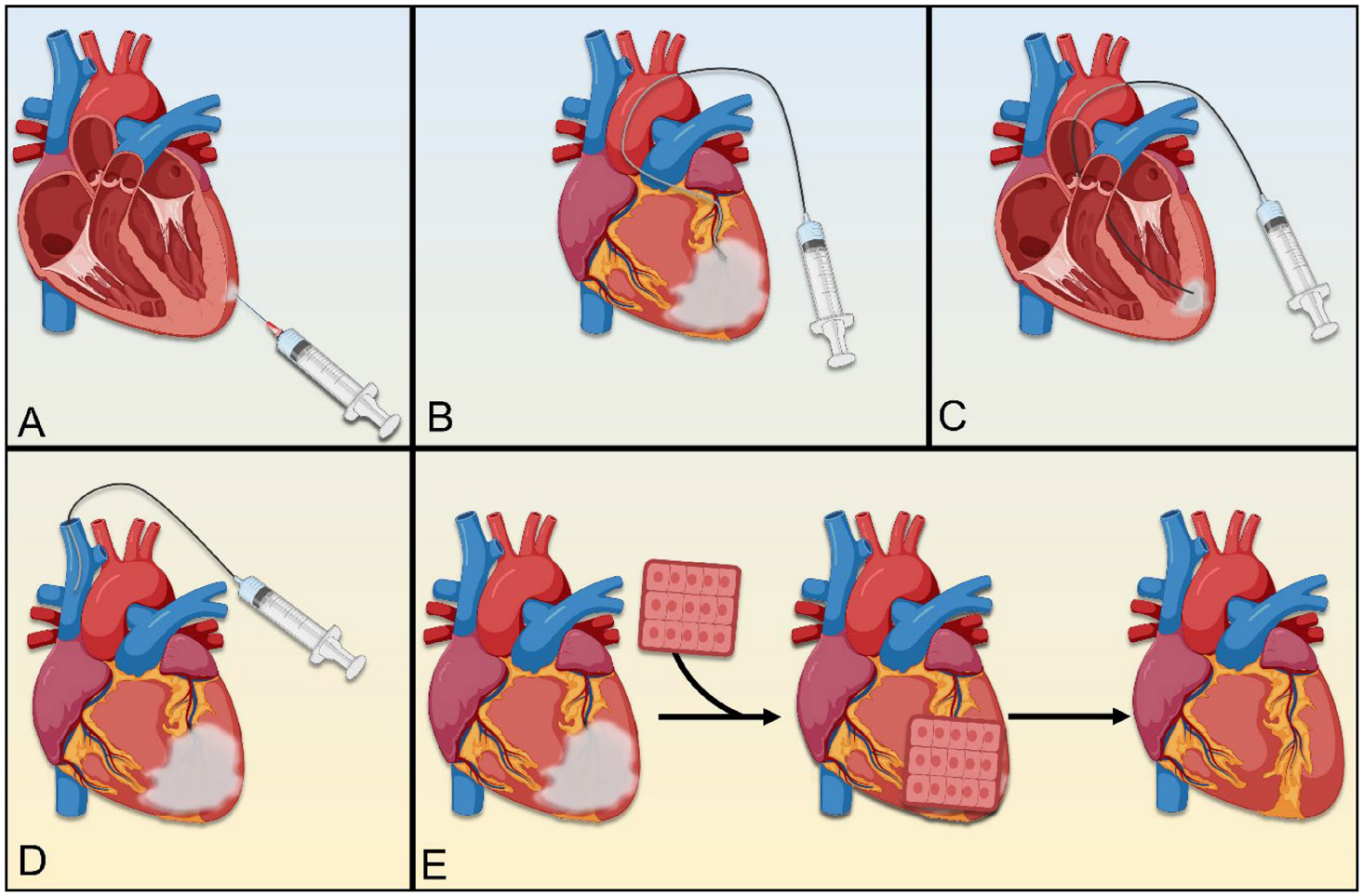
Different methods of stem cell administration into infarcted myocardium. These common routes of administration often lead to the limitations associated with stem cells for myocardial repair. (A) Epicardial injection of stem cells; (B) intra‐coronary injection of stem cells; (C) trans‐endocardial injection of stem cells; (D) intra‐venous injection of mesenchymal stem cells; (E) cell sheet administration of stem cells.

The most extensively studied stem cell types in cardiac repair are mesenchymal stem cells (MSCs) and induced pluripotent stem cell‐derived cardiomyocytes (iPSC‐CMs). MSCs are known for their immune‐privileged status and robust paracrine signalling, while iPSC‐CMs possess the unique ability to electrically couple with existing cardiomyocytes, thereby improving myocardial contractility [18, 19]. Notably, cardiac progenitor cells (CPCs) are stem‐like cells which also have a regenerative potential for myocardial repair [20]. Despite early enthusiasm, the clinical translation of SCT has encountered significant limitations (Figure 1) as large clinical trials demonstrated that SCs alone are not sufficient to elicit significant therapeutic benefit. One major issue is poor stem cell homing and retention in target areas [21]. After delivery, stem cells often struggle to survive in the hostile ischaemic microenvironment of the infarcted heart, which is rich in reactive oxygen species (ROS), proinflammatory cytokines, and hypoxic stress [19]. This leads to low engraftment rates and limited therapeutic effects. This insufficiency also appears to be affected by the route of delivery and origin of the SCs utilised [22]. For example, intracoronary injections of bone marrow‐derived SCs were shown to be problematic, particularly because both cell delivery and SCs' ability to migrate to injured myocardial areas were impaired by the high pressure of the coronary arteries and the blood flow therein (Figure 2) [17, 23].

Compounding these issues, the invasive nature of stem cell delivery—whether through intracoronary or intramyocardial injection—adds procedural risk. Even when successfully administered, many cells die shortly after injection, necessitating repeated administrations that further increase the risk to the patient [24]. There are also immunological challenges: allogeneic stem cell transplantation carries the potential for immune rejection due to major histocompatibility complex (MHC) mismatches, often requiring immunosuppressive therapy that increases susceptibility to infections [25]. Additionally, stem cells are prone to senescence during in vitro expansion, limiting their proliferation capacity, which is another obstacle given that large stem cell quantities are needed for therapeutic efficacy [26]. Notably, in the case of iPSCs, there is also a serious concern of teratoma formation due to residual undifferentiated cells capable of uncontrolled growth [25].

These challenges have led to an increasing interest in exosomes, a novel cell‐free therapeutic platform that could replicate many of the beneficial effects of SCT while overcoming its limitations. In fact, the limited therapeutic effects observed with stem cell injections have been largely attributed to exosomes secreted by the SCs themselves, which have recently gained attention for their reparative functions [27]. Exosomes are nano‐sized extracellular vesicles (40–160 nm) released by virtually all cells. Initially, exosomes were believed to function primarily in cellular waste disposal, but they are now being recognised as powerful mediators of intercellular communication and paracrine signalling [28, 29]. Indeed, exosomes carry bioactive cargo, including proteins, lipids, and nucleic acids such as microRNAs, that can alter the behaviour and fate of recipient cells. Notably, by virtue of their cardioprotective cargo, stem cell‐derived exosomes can reproduce many of the regenerative effects of their parent cells, such as promoting angiogenesis, reducing inflammation, suppressing fibrosis, and activating endogenous repair pathways [30, 31, 32, 33]. More importantly, exosomes overcome many of the key drawbacks associated with stem cell therapy. Compared to whole cells, exosomes have low immunogenicity, thereby minimising the risk of immune rejection and negating the need for immunosuppressants [34]. Additionally, their lipid bilayer confers stability and protection, allowing exosomes to circulate in the body without rapid degradation, and their small size enables them to cross biological barriers and reach target tissues more efficiently [34]. Moreover, exosomes do not replicate or transform, which eliminates the risk of teratoma formation, a concern that significantly hinders iPSC applications [35]. Intriguingly, exosomes can be bioengineered to enhance their targeting ability and therapeutic potency, including loading them with specific RNAs or drugs for precision delivery [36].

Given their biological stability, low immunogenicity, and strong therapeutic potential, exosomes represent a promising next‐generation alternative to traditional stem cells in the context of myocardial regenerative therapy. Among the various exosomes implicated in cardiac repair, exosomes derived from cardiac progenitor cells (CPCs), mesenchymal stem cells (MSCs), and, to a lesser degree, induced pluripotent stem cells (iPSCs) receive the lion's share [37]. Collectively, their main cardioprotective activities involve anti‐apoptotic, pro‐angiogenic, and post‐reperfusion repair in the ischaemic heart. In this light, the aim of this review is to discuss the main therapeutic roles of these stem cell‐derived exosomes in regenerative therapy following ischaemic injury.

## 2 Exosomes and microRNAs: Biogenesis, Function and Therapeutic Promise in Myocardial Infarction

Previously known as the ‘garbage bags’ of cells, exosomes have gained significant attention due to their important role in intercellular communication [38]. They are the smallest category of EVs released by all types of cells, and they act to deliver major biomolecules such as proteins, lipids and nucleic acids. Exosomes are detected within blood, saliva, and urine, among other physiological fluids [39, 40]. Reaching up to 160 nm in size, these nanoparticles protect their cargo against acid, RNAses, and heat by virtue of their phospholipid bilayer integrity [41]. Unlike other EVs, exosomes are solely endosomal in origin [41, 42].

**FIGURE 3:**
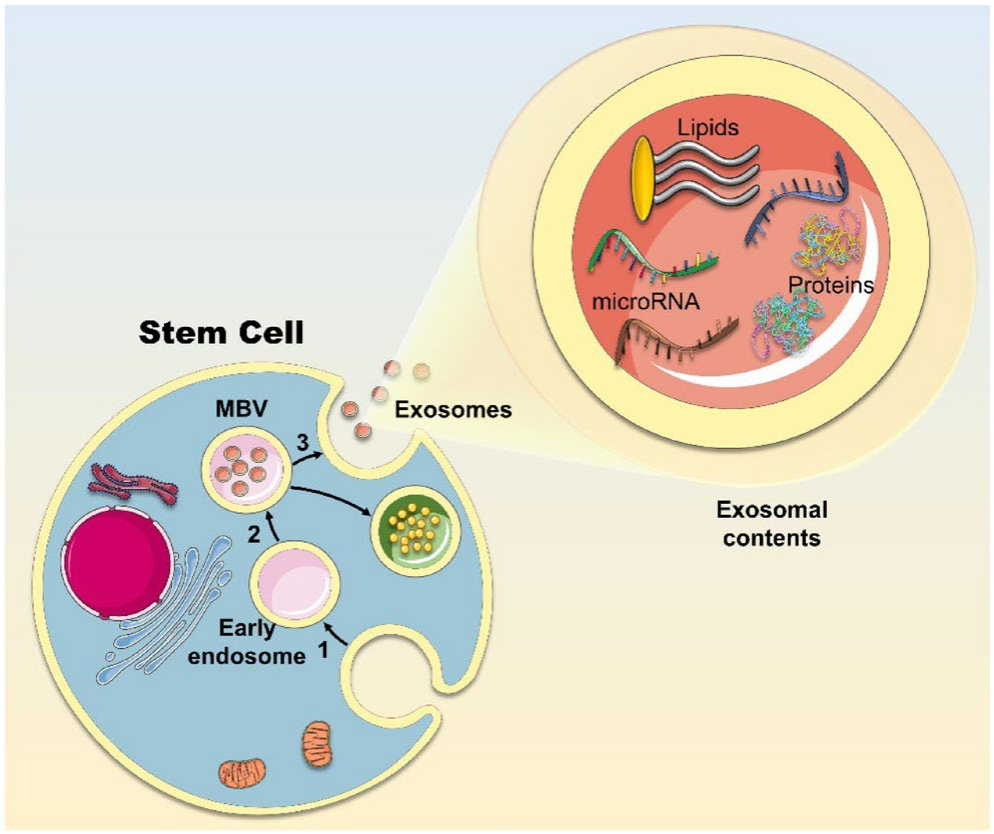
Exosome formation and contents. (1) The cellular membrane forms inward buds which pinch off to form endosomes. These vesicles undergo further inward budding, leading to the formation of intraluminal vesicles (ILVs) enclosed within the endosome, which is considered the multivesicular body (MVB) [43]. (2) During the process of ILV formation, cargo, including microRNA, lipids and proteins, is enclosed within the ILVs. (3) Eventually MVBs fuse with lysosomes to undergo degradation, or with the cell membrane, leading to the release of the enclosed nanovesicles known as exosomes.

In the initial phase of exosome formation, the plasma membrane buds inwardly and eventually pinches off, forming endosomes that undergo further inward invaginations by multiple mechanisms. This forms intraluminal vesicles (ILVs), which are enclosed within the endosome, now known as the multivesicular body (MVB) [43]. During the process of ILV formation, the cargo, encompassing membrane and cellular proteins as well as microRNAs (miRs), is sorted by a process suggested to be non‐random. Ultimately, the MVBs may fuse with either lysosomes for degradation or the plasma membrane for the release of the enclosed ILVs, now mature ‘exosomes’, into the extracellular space (Figure 3). Upon reaching target cells, exosomes communicate either via ligand‐cell membrane communication, fusion with the plasma membrane, or become endocytosed [44].

One of the most abundant entities transported by exosomes is microRNA (miR), a type of non‐coding RNA (ncRNA), usually 18 to 28 nucleotides long, and mostly implicated in epigenetic regulation of gene expression [45, 46]. MiRs are transcribed from their own genes or introns and exons into primary miR transcripts [47]. Once they reach the cytosol, they are cleaved into mature single‐stranded miRs and subsequently couple with the RNA‐induced silencing complex (RISC). Guiding RISC to the 3′ untranslatable region (UTR) of a target mRNA, the miR binds with partial or complete complementarity via its 2–8 nucleotide 5′ seed sequence [48], which then silences the mRNA translation, thereby rendering miRs the most copious category of epigenetic regulators [47].

**FIGURE 4:**
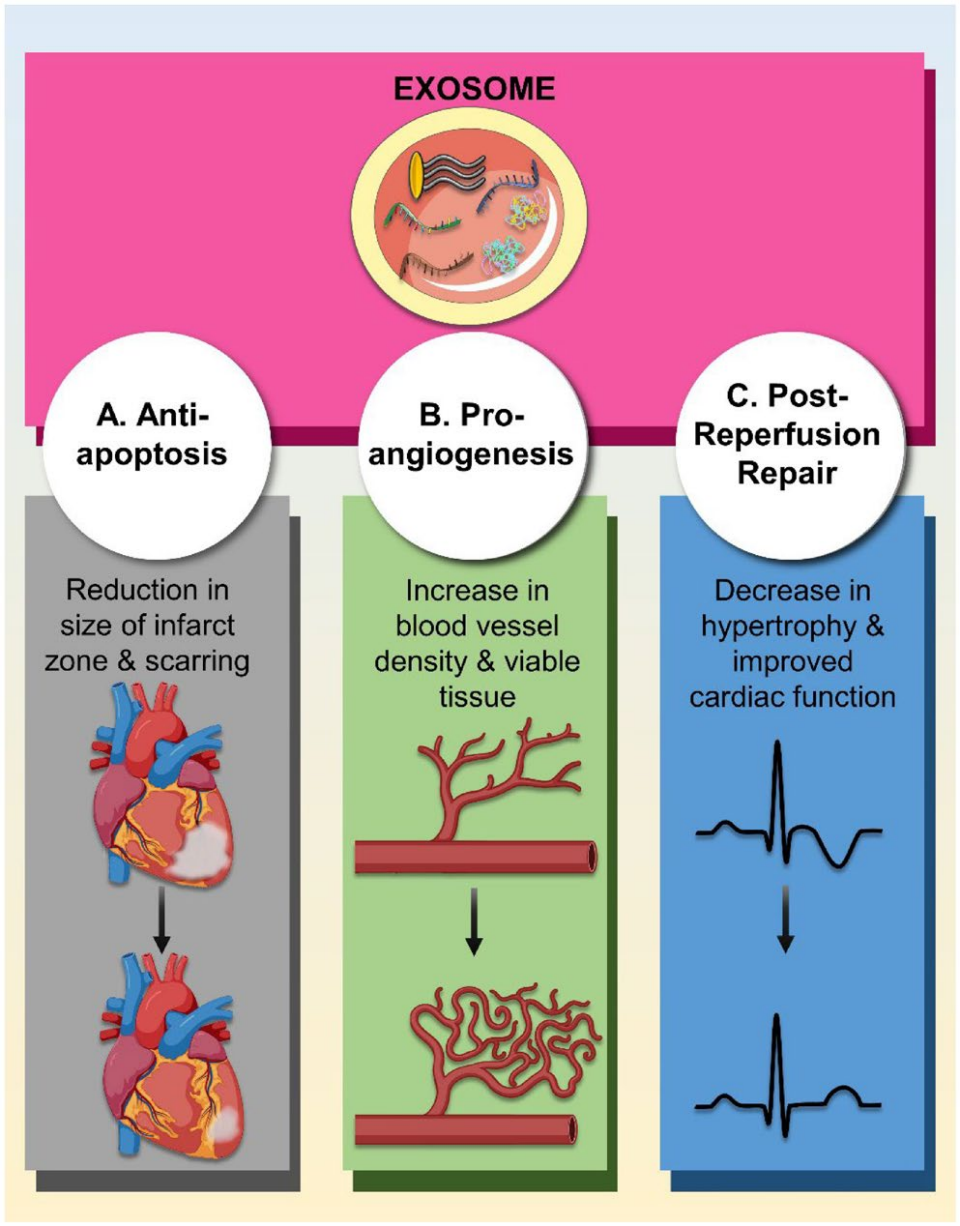
Major benefits from the use of stem‐cell derived exosomes post‐myocardial infarction. Anti‐apoptotic and pro‐angiogenic activity, as well as post‐reperfusion repair, are common advantages of using exosomes from various stem cell types for treatment after myocardial infarction.

In this context, it is now established that translation of over 60% of protein‐coding genes is regulated by microRNAs [39]. It is not surprising then that miRs regulate several important processes such as metabolism, immunity, differentiation, repair and apoptosis [49, 50, 51, 52]. Owing to their numerous functional roles, miR‐laden exosomes offer a large therapeutic potential for a plethora of diseases including myocardial ischaemia. Exosomal miRs are critical regulators of cardiac function, influencing cardiomyocyte survival, proliferation, regeneration, and differentiation in both physiological and pathological contexts [53]. For example, miR‐133 plays an essential role in cardiac development and homeostasis [54]. MiR‐1 regulates cardiac hypertrophy and electrical conduction, while miR‐133 supports cardiomyocyte proliferation and differentiation [55, 56]. Likewise, miR‐208a, a cardiac‐specific microRNA, is critical for maintaining cardiac contractility and conduction [57]. In the setting of MI, the expression of several miRNAs becomes significantly altered, resulting in either protective or pathological effects depending on the specific molecular pathways they regulate [58]. Notably, exosomes derived from CPCs, MSCs, and, to a lesser extent, iPSCs, are particularly enriched in cardioprotective miRNAs and proteins [37]. These exosomal cargoes act on multiple fronts in the ischaemic heart, orchestrating anti‐apoptotic, pro‐angiogenic, and post‐reperfusion reparative responses that collectively support myocardial healing and functional recovery (Figure 4).

## 3 The Role of Cardiac Stem Cell‐Derived Exosomes in Myocardial Repair

### 3.1 Therapeutic Potential of Cardiac‐Derived Stem Cells in Myocardial Repair

SCs are present throughout embryonic and adult life stages. Initially, embryonic SCs (ESCs) differentiate into cells that belong to any of the three germ lineages: endoderm, mesoderm and ectoderm [15]. Once committed to the lineage, tissue‐specific stem cells are formed, which can later give rise to one or more particular cell types.

In the heart, cardiac progenitor cells (CPCs) are stem‐like cells that give rise to numerous cardiac lineages [59], including cardiac resident cells, such as stem cell antigen‐1+ (Sca‐1+), side population (SP) cells, and cardiospheres (CS), among others [60]. These diverse populations of CPCs are found in different regions of the heart and can be identified and isolated at various stages of development based on their expression of specific surface and genetic markers. Notably, these cells are naturally located in the heart and exhibit potent regenerative properties, including proliferation, differentiation, and neovascularization [61]. Their intrinsic presence in cardiac tissue, combined with their regenerative capacity, has made them a compelling target for myocardial repair strategies, particularly in the setting of ischaemic heart disease (IHD).

Clinical interest in CPCs emerged alongside the broader field of cell‐based therapy for MI. Compared to extracardiac stem cells such as MSCs, cardiac stem cells exhibit stronger angiogenic potential, more efficient engraftment, and improved differentiation into cardiac lineages [62]. Among CPCs, several subtypes have been studied for their therapeutic potential in myocardial repair. c‐kit+ cells were the first identified and have shown modest improvements in cardiac function after MI, largely through paracrine effects rather than differentiation [63]. Conversely, KDR+/PDGFR‐α+ cells are capable of differentiating into CMs and vascular cells, contributing to reduced fibrosis and enhanced regeneration [64]. Sca‐1+ cells also display cardiogenic potential and self‐renewal properties but are primarily limited to animal models [64]. Other CPC subtypes such as cardiosphere‐derived cells (CDCs) have shown promising immunomodulatory and regenerative effects in both autologous and allogeneic settings, though their benefits likely arise from secreted factors rather than direct cardiomyocyte formation [65]. Side population cells (SPCs) and cardiac fibroblasts (CFs) have also been evaluated. SPCs can differentiate into CM‐like cells under specific conditions and have been shown to migrate to ischaemic regions and promote repair [66]. CFs, though traditionally considered structural support cells, can influence cardiac function via cytokine secretion and may differentiate into CM‐like cells following treatment with epigenetic modifiers [67, 68]. Collectively, these diverse CPC subtypes underscore the multifaceted potential of cardiac stem cells in myocardial repair, primarily through paracrine signalling and, to a lesser extent, direct differentiation into functional cardiac tissue.

Experimental and early clinical data support the role of cardiac stem cells (CSCs) in cardiac regeneration. A meta‐analysis of preclinical studies revealed that CSC therapy has been shown to improve left ventricular ejection fraction (LVEF) in small animal models by up to 12% [69]. More importantly, two clinical trials—SCIPIO and CADUCEUS—have evaluated the preliminary efficacy of c‐kit+ CSCs and CDCs, respectively. SCIPIO reported a progressive improvement in LVEF, while CADUCEUS showed structural improvements such as scar size reduction [70, 71]. These findings highlight the translational promise of CSC‐based therapies, demonstrating functional and structural benefits in early‐phase clinical trials.

#### 3.1.1 Limitations of Cardiac‐Derived Stem Cell Therapy and the Rise of CPC‐Derived Exosomes (CPC‐Ex) in Myocardial Repair

Despite their promising regenerative potential, the clinical application of CPCs faces several limitations. Although CPCs can proliferate, differentiate into various cardiac lineages, and promote neovascularization, their endogenous population in the heart is extremely limited, and isolating them poses logistical challenges [72]. More importantly, CPCs display poor viability and weak adhesion when transplanted into the infarcted myocardium, particularly due to the inflammatory and fibrotic microenvironment that impairs cell survival and engraftment [73]. Another major concern associated with CPC therapy is safety. Studies have reported risks of tumorigenicity and arrhythmogenesis, particularly when cells are immature or poorly differentiated [74, 75]. Notably, the solid three‐dimensional structure of cardiospheres raises the risk of embolism if administered via intracoronary or intravenous routes [76], prompting the need for alternative delivery methods such as pericardial injections (Figure 1).

CPCs also exhibit biological limitations, including susceptibility to senescence, decreased telomerase activity, and increased apoptosis, which limit their long‐term reparative capacity, especially in chronic infarcts [77]. Another notable challenge is immune rejection, particularly in the setting of allogenic CPC therapy. Although autologous CPC therapies offer a potential solution to this issue, they are costly, time‐consuming to prepare, and typically unavailable during the acute phase of MI, when rapid intervention is essential [73]. Essentially, the therapeutic efficacy of transplanted CPCs is often compromised by poor cell survival, limited migration to the injury site, and inadequate differentiation into mature cardiomyocytes [73]. As a result, direct tissue regeneration is minimal. Instead, many of the observed benefits of CPC therapy are increasingly attributed to their paracrine effects, whereby transplanted cells secrete exosomes containing bioactive factors that modulate inflammation, promote angiogenesis, and support endogenous repair processes [73].

These findings have fueled interest in using CPC‐derived exosomes as an alternative to CPC therapy in myocardial repair. These nanovesicles carry a cargo of proteins, lipids, and nucleic acids, notably cardioprotective microRNAs, and serve as critical mediators of intercellular communication in the heart. By delivering these functional biomolecules to cells in the infarcted myocardium, CPC‐ex can recapitulate the beneficial paracrine effects of CPCs while overcoming key drawbacks of whole‐cell transplantation. Unlike cell‐based therapies, exosomes are cell‐free and non‐replicative, which eliminates the risk of tumorigenesis or ectopic tissue formation [78]. Their acellular nature also simplifies storage and quality control, making them more amenable to clinical standardisation and large‐scale manufacturing. Moreover, CPC‐ex have low immunogenicity, which reduces the risk of immune rejection [78]. They also bypass the risk of arrhythmias associated with the engraftment of immature or heterogeneous cardiomyocytes, as they do not electrically integrate with host myocardium. Additionally, their small size and stability in circulation improve delivery efficiency and tissue penetration, overcoming many of the logistical challenges posed by cell transplantation [79]. Together, these features position CPC‐ex as a safer, more versatile alternative to traditional cardiac‐derived stem cell therapy in the context of myocardial repair.

### 3.2 Therapeutic Mechanisms of CPC‐Derived Exosomes in Myocardial Repair

Cardiac progenitor cell‐derived exosomes (CPC‐ex) have emerged as a promising acellular therapy for myocardial infarction, offering targeted and multifactorial mechanisms of repair [80]. These nanosized vesicles mediate their therapeutic effects primarily through the delivery of regulatory microRNAs and bioactive proteins that modulate key cellular processes involved in cardiac injury and healing. The following subsections detail the major functional roles of CPC‐ex in myocardial repair, focusing on their anti‐apoptotic activity, pro‐angiogenic effects, and post‐reperfusion repair.

#### 3.2.1 CPC‐Ex and Anti‐Apoptotic Activity

CPC‐ex play a crucial role in reducing cardiomyocyte apoptosis, a central process in myocardial injury following infarction. This cardioprotective function is primarily mediated by microRNAs contained within the exosomes, particularly miR‐21 and miR‐210. These miRs regulate apoptotic pathways by targeting pro‐apoptotic genes and activating survival signalling cascades.

MiR‐21 is one of the most prominent anti‐apoptotic miRs found in CPC‐ex. Incubation of rat H9C2 cardiomyoblasts with murine CPC‐ex for 24 h prior to the induction of apoptosis resulted in a significant reduction in the activity of caspases 3 and 7, key enzymes involved in the programmed cell death or apoptotic cascade [81, 82]. These effects have been attributed, in part, to miR‐21, which targets the transcript of programmed cell death 4 (pdcd4) in hypoxia‐exposed H9C2 cardiomyoblasts, thereby suppressing apoptosis and promoting cell survival [83]. Interestingly, while hypoxic conditions induced apoptosis in H9C2 cardiomyoblasts, CPCs subjected to hypoxia produced a greater quantity of exosomes. More importantly, these exosomes also showed a five‐fold increase in miR‐21 expression [20, 83]. This suggests that harvesting of exosomes from CPCs under hypoxic conditions may result in more copious quantities of therapeutic exosomal miRs; however, this remains to be fully and more carefully investigated. Collectively, these findings highlight miR‐21 as a key contributor to the anti‐apoptotic effects of CPC‐derived exosomes and suggest that modulating culture conditions, namely inducing hypoxia, could enhance their therapeutic potential in myocardial repair.

Beyond their effects on cardiomyocytes, miR‐21 has exhibited anti‐apoptotic properties in c‐kit+ cardiac stem cells by inhibiting PTEN, a known negative regulator of the PI3K/Akt signalling cascade [84]. By modulating the PTEN/PI3K/Akt pathway, miR‐21 enhanced cardiac stem cell proliferation and survival in vitro. Hence, miR‐21 may potentially serve as an adjunct to improve myocardial repair in cardiac‐derived stem cell therapy, which is often limited by low numbers and poor cell viability in the ischaemic myocardium. Yet, further investigation is warranted to elucidate the potential role of miR‐21 in stem cell therapy and myocardial repair.

MiR‐210 is among the most abundant miRs within CPC‐ex and plays a key role in their anti‐apoptotic effects. In animal models of MI, direct intra‐myocardial injection of miR‐210 resulted in a significant reduction in cardiomyocyte apoptosis [85]. Mechanistically, miR‐210 directly inhibits the translation of the pro‐apoptotic protein tyrosine phosphatase 1B (PTP‐1B) [80]. This inhibition has been shown to decrease levels of caspases 3 and 8 in cardiomyocytes, thereby reducing apoptosis. Furthermore, PTP‐1B suppression has been hypothesized to activate the PI3K/Akt signalling pathway in cardiomyocytes, promoting further cell survival and improved myocardial function [80, 86]. While these findings are promising in preclinical models, further research is needed to validate the therapeutic effects of miR‐210 in human subjects and to determine its safety and efficacy in clinical settings.

Supporting this translational potential, CPC‐ex have interestingly demonstrated cross‐species functionality. Not only were murine CPC‐ex bioactive in cardiac cells, but human‐derived CPC‐ex were also reparative in murine HL‐1 cardiomyocytes, wherein apoptosis was reduced by approximately 30% after exosome treatment [80]. This effect was also concomitant with a reduction in the activity of caspases 3 and 7 [80]. Similar dose‐dependent effects on suppressing apoptosis were also noticed in vivo when CPC‐ex were injected into the infarct border zone of the myocardium in rats with induced MI (Table 1) [80]. Exosomes derived from fibroblasts could not produce the same aforementioned effects, indicating that the CPC source and dose are important determinants of exosome anti‐apoptotic activity [80]. Building on these observations, a direct comparison between CPC‐ex and bone marrow‐derived mesenchymal stem cell‐derived exosomes (BM‐MSC‐ex) further underscores the superior cardioprotective potential of CPC‐ex [31]. In vitro experiments demonstrated that CPC‐ex significantly outperformed BM‐MSC‐ex in protecting HL‐1 cardiomyocytes from staurosporine‐induced apoptosis, showing consistent effects across a wide concentration range (10^5^–10^9^ particles/cm^2^). In contrast, BM‐MSC‐ex exhibited a much narrower therapeutic window, with notable anti‐apoptotic effects only observed at a single intermediate dose. These functional differences extended into angiogenesis assays, where both CPC‐ and BM‐MSC‐ex stimulated endothelial tube formation, but CPC‐ex exerted significantly stronger effects [31]. In vivo validation further strengthened these findings. When administered after permanent coronary occlusion in rat models, CPC‐ex resulted in more pronounced improvements in left ventricular function and a greater reduction in infarct size compared to BM‐MSC‐ex [31]. These results align with the mechanistic insights gained from proteomic profiling, which revealed a unique enrichment of active pregnancy‐associated plasma protein‐A (PAPP‐A) on CPC‐ex membranes. This protease mediates the release of insulin‐like growth factor‐1 (IGF‐1) by cleaving its binding protein (IGFBP‐4), thereby activating IGF‐1R signalling and downstream pro‐survival pathways such as Akt and ERK1/2 [101, 102, 103]. Collectively, these findings suggest that CPC‐derived exosomes not only offer broader and more potent effects on cardiomyocyte survival and angiogenesis compared to their BM‐MSC counterparts, but also act through distinct protease‐dependent mechanisms that directly modulate survival signalling. These differences support the notion that the source of exosomes plays a critical role in determining therapeutic efficacy, with CPC‐exosomes emerging as a more promising candidate for myocardial repair.

**TABLE 1:** Effect of various stem cell‐derived exosomes on reparative functions following myocardial infarction.

Type of stem cell‐derived exosome	Description of specific function	References
CPC‐exosomes		
I. Anti‐apoptotic activity (in vivo)	Injection of human CPC‐ex into the infarct border zone of mouse model of myocardial infarction (MI) reduced size of infarct zone and scarring (300 μg CPC‐Ex, by Day 7, *p* < 0.05)	[80]
Anti‐apoptotic exosomal microRNA	miR‐21 inhibits programmed cell death 4 (*pcd4*) transcript, resulting in decreased active caspase 3 and 7	[83]
	miR‐210 inhibits protein tyrosine phosphatase (PTP1), resulting in reduced apoptosis	[80]
Effect of hypoxia on exosomes	Increased quantity of CPC‐ex production and upregulation of miR‐21	[83]
II. Pro‐angiogenic activity (in vivo)	Injection of human CPC‐ex into the infarct border zone of mouse model of myocardial infarction (MI) increased blood vessel density and viable tissue (300 μg CPC‐Ex, by Day 7, *p* < 0.05)	[80]
Pro‐angiogenic exosomal microRNA	mir‐132 downregulates expression of RasGap‐p120, leading to increased pro‐angiogenic activity	[80]
	miR‐20 inhibits the anti‐angiogenic proteins ephrin A3 and PTP1	[80]
	miR‐322 enhanced angiogenesis partially via increased NOX2‐derived ROS	[87]
Effect of hypoxia on exosomes	Increased quantity of CPC‐ex production and enhanced reparative function. Tube formation was greater compared to CPC‐ex from normoxic conditions. CPC‐ex derived after 3 h of hypoxic conditions were ineffective compared to CPC‐ex obtained after 12 h of hypoxia exposure	[80]
III. Repair after post‐reperfusion injury (in vivo)	Human paediatric CPC‐ex (injected at a dose of 80 μg/kg body weight), regardless of age and culture oxygenation, decreased peri‐infarct hypertrophy (assessed at day 28 post‐injection) in rats	[88]
Post‐reperfusion repair exosomal microRNA	miR‐29c and miR‐27a exert anti‐fibrotic activity	[88]
	miR‐451 upregulated expression of COX‐2 reduced post‐reperfusion apoptosis	[81]
Effect of hypoxia on exosomes	Improved ejection fraction by hypoxic and normoxic CPC‐ex from day 3‐day 28	[88]
	Reperfusion‐associated apoptosis was reduced by 53% upon prior injection with 25 μL of CPC‐ex in mice	[81]
	Hypoxic CPC‐ex from older children improved EF and decreased scarring not before day 28	[88]
MSC‐exosomes		
I. Anti‐apoptotic activity (in vivo)	Injection of hypoxic BM‐MSC‐ex led to reduced apoptosis in heart tissue (7 days post‐treatment) as well as decreased apoptotic proteins such as BAX and caspase‐3	[89]
Anti‐apoptotic exosomal microRNA	miR‐210 was significantly upregulated in hypoxic exosomes and mediates its anti‐apoptotic effect partially by inhibiting PI3K/Akt pathway	[89]
	miR‐25‐3p‐mediated NFƘB inhibition and suppression of cytokine levels and pro‐apoptotic proteins	[90]
	MiR‐21 inhibits expression of the pro‐apoptotic protein PDCD4 (programmed cell death factor 4)	[91]
Effect of hypoxia on exosomes	Hypoxia significantly enhances exosome secretion from cultured MSCs	[89]
	Hypoxic BM‐MSC‐ex contained upregulated levels of miR‐210 compared to normoxic exosomes and they induced more pronounced cell viability in ischaemic cardiomyocytes	[89]
II. Pro‐angiogenic activity (in vivo)	Intramyocardial injection of mouse‐derived BM‐MSC‐ex into mice (600 μg) led to a significant increase in blood vessel density and cardiac function from day 7 to day 28	[92]
Pro‐angiogenic exosomal microRNA	Overexpression of miR‐132 in the exosomes led to significant downregulation of RASA1, causing an upregulation of RAS/MAPK signalling and downstream angiogenesis	[91]
	miR‐21 led to activation of ERK and protein kinase B (Akt), resulting in increased VEGF and HIF‐1 and subsequent angiogenesis	[93]
	miR‐1246 was shown to activate SMAD1/5/8 signalling in HUVECs, leading to angiogenesis	[94]
III. Repair after post‐reperfusion injury (in vivo)	Injection of BM‐MSC‐ex (5 μg dissolved in 10 μL PBS) into the peri‐infarct region 5 min prior to reperfusion, resulted in increased LC3B (autophagy protein) expression, reduced apoptosis, diminished infarct region and improved cardiac function 7 h post sacrifice	[95]
	Rat derived BM‐MSC‐ex (10 μg) delivered to rat models of infarction/reperfusion led to reduced apoptosis and less pronounced abnormal cell shape exosomes induced expression of Atg13 (stimulates autophagic activity via mTOR) and suppressed Apaf1 (a modulator of mitochondrial apoptotic processes)	[96]
	Intravenous delivery of human embryonic MSC‐ex (16 μg/kg body weight) into M.I‐induced mice prior to reperfusion therapy led to a reduced infarct size by approximately one half, within 1 day after reperfusion	[97]
Post‐reperfusion repair exosomal microRNA	Post reperfusion repair was attributed to the exosomal miR‐19a, −21, −22, −24, −125b, −144, which suppress expression of apoptotic genes (like bcl2) while activating anti‐apoptotic and pro‐survival pathways mediated by PI3K/Akt/mTOR	[95]
Effect of hypoxia on exosomes	Hypoxia increases production of BM‐MSC‐derived exosomes (BM‐MSC‐ex) and stimulates their uptake by H9c2 cells	[98]
iPSC‐exosomes		
I. Post‐reperfusion repair (in vivo)	Intra‐myocardial injection of iPSC exosomes (100 μg) post‐reperfusion therapy in mice displayed improved cardiac function (lowered LVEF) and cardiac tissue repair by day 35	[99]
Reparative exosomal microRNA	Injection of iPSC‐ex (25 μL) after induction of M.I in mice significantly reduced apoptosis by approximately 70%, which was attributed to a decrease in caspase‐3 activity (24 h after reperfusion)	[100]
	miR‐21 and miR‐210, the latter of which is established to reduce oxidative stress and apoptosis	[100]

Taken together, these findings highlight the roles of miR‐21 and miR‐210 in mediating the anti‐apoptotic effects of CPC‐derived exosomes, which act through distinct yet complementary molecular mechanisms. By targeting key pro‐apoptotic regulators such as PDCD4 and PTP‐1B and enhancing survival pathways like PI3K/Akt, these miRs have shown their ability to contribute to improved cardiomyocyte viability both in vivo and in vitro. The ability of CPC‐ex to exert therapeutic effects across species and their superiority to BM‐MSC‐ex further underscores their potential as a novel and versatile tool in cardiac regenerative medicine. However, rigorous clinical studies are essential to validate these findings in humans and to optimise their safety, dosing, and delivery in future therapeutic applications.

#### 3.2.2 CPC‐Ex and Pro‐Angiogenic Activity

Angiogenesis is necessary to re‐establish proper cardiac function after MI. In this context, CPC‐ex were shown to effectively induce angiogenesis in both cell and animal models. These pro‐angiogenic effects are mediated through multiple mechanisms driven by the enriched content of CPC‐ex, which includes key pro‐angiogenic microRNAs (miR‐132, miR‐210, and miR‐322) along with angiogenic proteins such as vascular endothelial growth factor (VEGF), matrix metalloproteinase‐9 (MMP‐9), and extracellular matrix metalloproteinase inducer (EMMPRIN) [Table 1].

Among the most critical pro‐angiogenic miRNAs in CPC‐ex is miR‐132, which enhances endothelial tube formation by suppressing RasGAP‐p120, a negative regulator of the Ras/ERK signalling pathway [80]. By downregulating RasGAP‐p120, miR‐132 enhances Ras/ERK activation, thereby promoting endothelial cell proliferation and migration. These effects have been validated in vitro where human umbilical vascular endothelial cells (HUVECs) incubated with human CPC‐ex developed endothelial tubes, indicative of angiogenesis in vitro [80]. In tandem, injection of CPC‐ex into the infarct border zone in animal models caused a dose‐dependent increase in blood vessel density (angiogenesis) and viable tissue within 7 days (Table 1) [80]. Collectively, these findings underscore the role of miR‐132 in mediating the pro‐angiogenic effects of CPC‐ex, positioning it as a key molecular driver of vascular regeneration following myocardial injury.

MiR‐210 is another highly enriched microRNA in CPC‐ex which further amplifies its angiogenic effects. Mechanistically, miR‐210 exerts its pro‐angiogenic effect primarily by directly inhibiting the translation of the anti‐angiogenic protein, ephrin A3 (Efna3) [80]. Additionally, miR‐210 downregulates PTP1B, a phosphatase that negatively regulates VEGF signalling [73]. By suppressing these two factors, miR‐210 effectively enhances VEGF‐mediated endothelial cell activation and neovascularization. Notably, in vivo delivery of miR‐210 into the peri‐infarct region significantly improved neovascularization within 4–8 weeks post‐treatment, reinforcing its crucial role in CPC‐ex‐mediated vascular regeneration [85].

The pro‐angiogenic effects of CPC‐ex are also mediated by miR‐322, which boosts NADPH oxidase‐2 (NOX2)‐derived ROS in endothelial cells [87]. While high ROS levels are typically deleterious, controlled NOX2‐derived ROS act as signaling molecules to promote angiogenesis by enhancing endothelial proliferation, migration, and tubulogenesis [104]. Interestingly, bioengineered CPC‐ex transfected with pro‐angiogenic miR‐322 (CPC‐ex‐322) demonstrated therapeutic efficacy in a mouse model of MI [87]. Compared to unmodified CPC‐ex, systemic delivery of CPC‐ex‐322 conferred superior therapeutic benefits, including enhanced angiogenesis and improved protection of the infarcted myocardium. Notably, CPC‐ex‐322 administration led to a significant increase in blood vessel density within the border zones of infarcted hearts, confirming the functional relevance of miR‐322 in post‐MI vascular regeneration [87]. These findings suggest that bioengineering CPC‐ex through cardioprotective microRNA programming represents a promising strategy to enhance the therapeutic efficacy of CPC‐ex in ischaemic heart disease.

Beyond their microRNA cargo, CPC‐ex also contains potent pro‐angiogenic proteins that contribute to their therapeutic efficacy. Among the most notable are VEGF, MMP‐9, and EMMPRIN, each of which plays a critical role in facilitating vascular regeneration following myocardial injury [72]. VEGF is a well‐established mediator of angiogenesis that promotes endothelial cell proliferation, migration, and new vessel formation [105]. MMP‐9, a matrix‐degrading enzyme, enhances this process by remodeling the extracellular matrix (ECM), thereby allowing endothelial cells to migrate and invade surrounding tissue [106]. EMMPRIN acts upstream by stimulating the production and secretion of MMPs from neighbouring stromal and endothelial cells [107]. Together, MMP‐9 and EMMPRIN form a synergistic pair that reshapes the myocardial microenvironment to support neovascularization. Importantly, functional studies have supported the essential role of these CPC‐ex protein cargoes [108]. Human CPC‐ex have been shown to significantly enhance endothelial proliferation, tube formation, and migration in vitro. Notably, knockdown of key proteins such as EMMPRIN resulted in a marked reduction in angiogenesis both in vitro and in vivo [109]. Taken together, these findings demonstrate the role of CPC‐ex pro‐angiogenic protein cargo, which enhances vascular regeneration in the context of MI.

Ultimately, CPC‐ex orchestrates pro‐angiogenic effects through multifaceted mechanisms involving both microRNA and protein cargo. By delivering miR‐132, miR‐210, and miR‐322, alongside pro‐angiogenic proteins such as VEGF, MMP‐9, and EMMPRIN, CPC‐ex modulates key molecular pathways that promote endothelial proliferation, migration, and vascular remodelling. These synergistic effects position CPC‐ex as a potent therapeutic tool for restoring perfusion and supporting myocardial repair after ischaemic injury.

#### 3.2.3 CPC‐Ex and Post‐Reperfusion Repair

One of the key challenges in the management of MI is the paradoxical injury caused by reperfusion itself. While reperfusion is essential to restore blood flow, the sudden reintroduction of oxygen into previously ischaemic myocardium generates a burst of ROS, which triggers mitochondrial dysfunction, oxidative stress, and apoptotic signalling—collectively known as ischaemia–reperfusion (I/R) injury [110]. Notably, CPC‐ex have demonstrated the ability to mitigate this reperfusion‐associated cell death and preserve myocardial function. These cardioprotective effects are driven by a coordinated network of regulatory microRNAs (miR‐451 and miR‐146a) enriched within CPC‐ex.

A key mediator of the post‐reperfusion cardioprotective effects of CPC‐ex is miR‐451, which is highly enriched in CPC‐ex. In murine models of I/R injury, intramyocardial injection of CPC‐ex immediately before reperfusion resulted in a significant 53% decrease in cardiomyocyte apoptosis within 24 h [Table 1] [81]. Mechanistically, miR‐451 exerts anti‐apoptotic effects by inhibiting the activation of caspase‐3 and caspase‐7, key executors of programmed cell death. Interestingly, hypoxic stress activates the cardiac‐specific transcription factor GATA‐4, which induces the expression of miR‐451 [111]. Subsequently, this microRNA upregulates cyclooxygenase‐2 (COX‐2), which in turn activates the prostaglandin E2 (PGE2) survival pathway, contributing to cardiomyocyte resistance against reperfusion‐induced injury [111, 112]. Moreover, overexpression of COX‐2 in cardiomyocytes reduces the availability of unesterified arachidonic acid, a substrate known to exacerbate reperfusion injury through multiple pathways [113]. During reperfusion, the surge in ROS promotes the non‐enzymatic peroxidation of arachidonic acid, resulting in the formation of 8‐iso‐prostaglandin F2α (8‐iso‐PGF2α)—a bioactive isoprostane known to exacerbate reperfusion injury [114]. In addition, excess arachidonic acid can directly impair mitochondrial function by inhibiting complexes I and II of the electron transport chain and by promoting the opening of the mitochondrial permeability transition pore (mPTP), both of which contribute to cardiomyocyte death and tissue damage during I/R injury [115, 116]. Hence, by decreasing the levels of unesterified arachidonic acid, COX‐2 confers cardioprotection by mitigating oxidative stress and mitochondrial dysfunction [117, 118]. Collectively, these findings highlight the role of the GATA‐4/miR‐451/COX‐2 axis in orchestrating a protective program that improves cardiomyocyte survival during reperfusion, thereby underscoring the therapeutic potential of CPC‐ex in MI [111].

Exosomes derived from CDCs, a subtype of CPCs, exert potent cardioprotective effects, largely through the action of miR‐146a. Notably, in a murine model of MI, this microRNA was shown to regulate inflammation, oxidative stress, and fibrosis, which are three key pathological processes implicated in I/R injury [119]. Mechanistically, miR‐146a targets interleukin‐1 receptor‐associated kinase 1 (IRAK1) and TNF receptor‐associated factor 6 (TRAF6), two critical adaptor proteins in the Toll‐like receptor (TLR) signalling pathway [120, 121]. By suppressing IRAK1 and TRAF6, miR‐146a blunts the TLR‐mediated activation of NF‐κB, thereby decreasing pro‐inflammatory cytokine production during MI [119]. Additionally, miR‐146a modulates oxidative stress by targeting NADPH oxidase 4 (NOX4), a major source of ROS in cardiomyocytes [122]. Suppression of NOX4 reduces oxidative damage during reperfusion, thereby preserving myocardial tissue integrity [123]. Moreover, miR‐146a downregulates SMAD4, a key effector in the TGF‐β signalling cascade, thereby attenuating fibrotic cardiac remodelling [124]. Collectively, these effects contribute to a more favourable microenvironment that supports myocardial healing following reperfusion injury. Interestingly, although exogenous delivery of miR‐146a alone reproduces some of these effects, such as increased viable mass and reduced inflammation, it fails to achieve the full functional and structural benefits of CDC‐ex, including significant scar reduction and improved cardiac output [119]. This observation suggests that additional miRNAs likely contribute synergistically to the overall therapeutic efficacy of CDC‐ex. For instance, miR‐22 has been implicated in promoting adaptive responses to cardiac stress [125], while miR‐24 has been shown to reduce cardiac fibrosis by targeting furin, a key activator in the profibrotic TGF‐β signalling cascade [126]. However, the potential roles of miR‐22 and miR‐24 as mediators of the cardioprotective effects of CDC‐ex in MI remain to be investigated. Nonetheless, these findings highlight the potential complex regulatory network of microRNAs within CDC‐ex, which collectively promote cardiac regeneration following myocardial injury.

Taken together, CPC‐ex offers a multifaceted approach to post‐reperfusion myocardial repair by mitigating apoptosis, inflammation, oxidative stress, and fibrosis through the coordinated actions of regulatory microRNAs, namely miR‐451 and miR‐146a. These findings highlight the potential of CPC‐ex as a powerful, cell‐free platform for improving cardiac function and post‐reperfusion repair in MI.

## 4 The Role of Mesenchymal Stem Cell‐Derived Exosomes (MSC‐Ex) in Myocardial Repair

### 4.1 Therapeutic Potential of Mesenchymal Stem Cells (MSCs) in Myocardial Repair

MSCs are fibroblast‐like multipotent cells that can be found in many tissues such as bone marrow, muscle, placenta, umbilical cord, and adipose tissue. Their availability from multiple sources and ease of access via minimally invasive methods make them an attractive option for therapeutic use [127]. Owing to their mesodermal origin, MSCs can differentiate into multiple cell types such as chondrocytes, osteocytes, and adipocytes. Additionally, they have also demonstrated the ability to differentiate into ectodermal lineages, giving rise to cardiomyocytes, smooth muscle and endothelial cells, making them promising candidates for cardiac repair [15, 60].

In the context of MI, MSCs have shown promising cardioprotective effects as evidenced by their ability to improve cardiac function and repair the injured site. These cells aggregate and cluster over the injured site where they secrete a broad spectrum of cytokines, growth factors, and microRNAs that collectively modulate inflammation, promote angiogenesis, and inhibit apoptosis [128]. Indeed, experimental studies have highlighted the therapeutic efficacy of MSCs. For example, MSCs overexpressing miR‐133 significantly enhanced cardiac function in rodent models of MI [129]. This cardioprotective effect was attributed to the anti‐apoptotic, anti‐inflammatory and anti‐fibrotic roles of this microRNA, thereby underscoring the therapeutic potential of MSCs in cardiac injury [129, 130]. Furthermore, since MSCs do not need to differentiate into more mature cells before administration, and due to the absence of histocompatibility class II molecules on their surface, they can evade both human and animal immune surveillance and have been shown to cause insignificant immunoreaction [60]. This minimises the risk of rejection and enhances the translational potential of MSC therapy. To optimise therapeutic delivery and retention of MSCs, various administration routes—including intravenous, intracoronary, transendocardial and epicardial injections—have been explored, alongside new emerging techniques such as injectable biomaterial scaffolds and cell sheets [131]. Adding to their therapeutic potential, MSCs are amenable to genetic modifications using viral vectors [60] and can be expanded in vitro while maintaining their phenotype and functionality. These properties collectively position MSCs as a valuable tool for myocardial repair by enhancing tissue regeneration, modulating the immune response, and limiting fibrotic remodelling.

#### 4.1.1 Limitations of MSC Therapy and the Rise of MSC‐Ex in Myocardial Repair

Despite these numerous advantages, the use of MSCs in the treatment of MI is limited by several factors (Figure 1). Although they have been found to adhere and facilitate angiogenesis and cardiomyogenesis at the site of injury, their rate of adherence and differentiation is considerably low after MSC transplantation [60]. In addition, MSCs were associated with immunosuppression after administration due to the presence of anti‐inflammatory cytokines in their environment. While this can be beneficial in reducing post‐infarction inflammation, this immunosuppression could be detrimental to the organism's protection against pathogens if it were to be extensive or uncontrolled [132]. Importantly, injection of MSCs is often followed by a rapid decline in their number, suggesting that the reduction in infarct size is likely not a direct result of MSC trans‐differentiation within the injured myocardium, but possibly due to their paracrine activity. In fact, it was shown that the culture medium of various MSCs enhances the survival of cardiomyocytes, stimulates angiogenesis, and reduces infarct size [133, 134].

Indeed, accumulating evidence suggests that the beneficial effects of MSCs are predominantly due to their secreted extracellular vesicles, particularly exosomes [37]. These nano‐sized vesicles carry bioactive molecules such as proteins, lipids, and regulatory RNAs that replicate the paracrine effects of their parent cells. Notably, recent advances have highlighted mesenchymal stem cell‐derived exosomes (MSC‐ex) as a promising alternative that can overcome many of the drawbacks associated with MSC therapy [135]. MSC‐ex preserve the beneficial paracrine effects of MSCs—such as immunomodulation, anti‐apoptotic activity, and promotion of angiogenesis—while avoiding the complications linked to the use of live cells [29]. The nanoscale size and membrane composition of exosomes allow them to traverse biological barriers, including capillary walls, enabling efficient delivery of their bioactive cargo—proteins, lipids, RNAs, and growth factors—to injured myocardial tissue [37]. Because exosomes are cell‐free and non‐replicative, they carry a markedly reduced risk of tumour formation or ectopic tissue development compared to cell‐based therapies [136]. The acellular nature of MSC‐ex simplifies production, storage, and handling, enhancing clinical scalability and standardisation [137]. Additionally, exosomes have demonstrated therapeutic efficacy across a wide range of settings, including cardiovascular diseases, and can be engineered to serve as natural delivery vehicles for targeted therapeutic molecules [138]. Taken together, these features underscore the superiority of MSC‐derived exosomes over traditional MSC therapies. By addressing key issues such as poor engraftment, limited survival and immune complications, while preserving therapeutic functionality, MSC‐ex represent a safer, more effective and clinically viable approach to myocardial repair.

### 4.2 Therapeutic Mechanisms of MSC‐Derived Exosomes in Myocardial Repair

MSC‐ex have emerged as potent cell‐free therapeutic agents capable of addressing key pathological processes in myocardial infarction and I/R injury. Their multifaceted reparative functions are mediated by a rich cargo of bioactive molecules—including microRNAs, proteins, and enzymes—that collectively target and modulate cardiomyocyte survival, angiogenesis, and tissue recovery. The following subsections explore three principal mechanisms by which MSC‐ex exert their cardioprotective effects, including anti‐apoptotic activity, pro‐angiogenic stimulation and post‐reperfusion repair.

#### 4.2.1 MSC‐Ex and Anti‐Apoptotic Activity

Exosomes constitute a major bioactive component of the MSC conditioned medium, and their experimental application has shown promising therapeutic results. Treatment of murine cardiomyocytes with mouse‐derived BM‐MSC‐ex caused a significant drop in induced‐apoptosis [90] by reducing cardiomyocyte levels of several pro‐apoptotic proteins and cytokines [90]. Mechanistically, MSC‐ex deliver a wide range of microRNAs that act in concert to suppress cardiomyocyte apoptosis through multiple molecular pathways. For instance, incubation of cardiomyocytes with BM‐MSC‐ex led to a significant increase in the level of miR‐25‐3p [90]. This miR exerts its protective effects by inhibiting IκB phosphorylation, thereby blocking activation of NF‐κB, a central mediator of inflammation and apoptosis. This mechanism demonstrates that MSC‐exosomes preserve cardiomyocyte viability by modulating inflammatory apoptotic signalling partially through miR‐25‐3p‐mediated NF‐kB inhibition [90]. Another miRNA which contributes to the anti‐apoptotic potential of MSC‐ex is miR‐21, which activates the PI3K/Akt pathway, thereby suppressing caspase‐3 and caspase‐7 activity and promoting cell survival during myocardial infarction [139]. Other examples include miR‐24, which inhibits mitochondrial apoptosis by targeting Bax, a pro‐apoptotic Bcl‐2 family member [140]. Moreover, miR‐19a inhibits JNK3/caspase 3 signalling, thereby inhibiting cardiomyocyte apoptosis and preserving cardiac function [141]. These miRNAs exhibit synergistic interactions, as they act on distinct but complementary pathways, amplifying the overall anti‐apoptotic efficacy of MSC‐exosomes. This synergy between miR‐25‐3p, miR‐21, miR‐24 and miR‐19a exemplifies the multi‐targeted action of MSC‐exosomes in protecting the ischaemic myocardium.

Beyond their miRNA cargo, MSC‐exosomes also carry bioactive proteins that contribute to their regulation of apoptosis. For instance, BM‐MSC‐exosomes contain itchy E3 ubiquitin ligase (ITCH), which has been shown to inhibit H9C2 cardiomyoblast apoptosis in acute myocardial infarction models. Mechanistically, ITCH promotes the ubiquitous degradation of apoptosis signal‐regulating kinase‐1 (ASK1), a key activator of the pro‐apoptotic JNK/p38 signalling pathway [142]. Therefore, inhibition of this pathway mitigates cardiomyoblast apoptosis and attenuates myocardial injury in acute MI. Another protein found in MSC‐exosomes is MFGE8 (milk fat globule‐EGF factor 8), which facilitates the clearance of apoptotic cells by linking phosphatidylserine on dying cells to integrins on phagocytes, thereby resolving inflammation in the injured myocardium and promoting tissue repair [143]. Taken together, these bioactive protein components in MSC‐exosomes add a secondary layer of anti‐apoptotic signalling that complements their miRNA cargo.

MSC‐exosomes derived from multiple tissue sources have demonstrated cardioprotective effects by targeting various apoptotic pathways. In an in vitro model of hypoxia‐conditioned HL‐1 cardiomyocytes, human gingiva‐derived MSC‐exosomes (hG‐MSC‐ex) decreased the expression of key apoptotic markers such as CASP3 and BAX, thereby enhancing cell survival [144]. Another study using exosomes from adipose‐derived MSCs (AD‐MSC‐ex) showed that they activate the Wnt/β‐catenin signalling pathway, which reduces myocardial apoptosis and supports cardiomyocyte viability following I/R injury in rats [145]. These findings suggest that the anti‐apoptotic effects of MSC‐exosomes are not restricted to a single tissue origin, but are a shared therapeutic feature across different MSC sources. This cross‐source consistency enhances the versatility and translational potential of MSC‐exosome therapy in myocardial infarction.

Taken together, these findings support MSC‐exosomes as potent anti‐apoptotic agents in myocardial injury. Through the delivery of various miRNAs and apoptosis‐regulating proteins, MSC‐exosomes preserve cardiomyocyte viability and reduce myocardial damage. This multifaceted protective capability, in addition to their numerous sources, positions MSC‐exosomes as a promising platform for cell‐free myocardial therapy.

#### 4.2.2 MSC‐Ex and Pro‐Angiogenic Activity

Angiogenesis emerged as one of the main reparative functions of MSC‐ex in the context of myocardial repair. Analysis of the miR profile of human BM‐MSCs‐ex indicated that the most expressed 23 miRs are predicted to regulate the expression of more than 5000 genes [146], many of which modulate angiogenesis. MSC‐ex are taken up by HUVECs in a time‐dependent manner, and at concentrations greater than 2.5 μg/mL, parameters indicative of increased angiogenesis, such as total tube length, are potentiated. This could be attributed to specific miRNAs carried by MSC‐ex, which orchestrate the molecular events that underlie angiogenesis in the injured myocardium. For example, miR‐205, delivered by adipose‐derived MSC‐ex, was shown to directly stimulate the proliferation and migration of microvascular endothelial cells, as evidenced by a significant upregulation of angiogenic markers, including HIF‐1α and VEGF [147]. Similarly, exosomal miR‐543 promoted cardiac microvascular endothelial cell (CMEC) angiogenesis after MI by downregulating COL4A1, a gene associated with arterial stiffness and ischaemia [148]. In addition, miR‐486‐5p was shown to mediate the angiogenic and cardiac reparative benefits of exosomes derived from hypoxia‐conditioned MSCs in both rodent and primate MI models [149]. These findings support the idea that certain exosomal miRNAs act cooperatively to regulate angiogenesis, and that exosome efficacy is closely tied to the content of their miRNA cargo.

ECs are important mediators of angiogenesis, and their uptake of BM‐MSC‐ex has been shown to aid this process. Mouse‐derived BM‐MSC‐ex were efficiently taken up by target HUVECs and led to significant upregulation of miR‐132, essential for significant in vitro and in vivo angiogenesis in HUVECs and mice, respectively [92]. Mouse models of MI, intramyocardial injection of BM‐MSC‐ex (600 μg) significantly increased blood vessel density and improved cardiac function (e.g., LVEF) as early as 7 days and continued to do so until day 28 (Table 1) [92]. This effect was enhanced when the exosomes enclosed overexpressed quantities of miR‐132. A mechanism by which miR‐132 exerts its effect is via downregulation of RASA1 (which encodes for the protein p120RasGAP) in target cells, which leads to downregulation of the RAS/MAPK pathway, thereby shunting angiogenesis. By suppressing RASA1, miR‐132 disables this inhibition and facilitates the formation of new blood vessels [91]. These studies collectively suggest that exosomes derived from MSCs offer promising pro‐angiogenic activity, largely via their library of miRs.

Interestingly, age‐related decline in angiogenic potential was linked to differential miRNA expression in MSC‐exosomes. For example, bone marrow MSC‐exosomes from aged donors showed reduced ability to promote endothelial tube formation and cardiac repair compared to those from younger donors [150]. This impairment was associated with downregulation of miR‐221‐3p, whose restoration recovered the MSC‐ex‐mediated pro‐angiogenic effects [150]. This highlights that not only the miRNA content but also the source and condition of MSCs play a critical role in determining exosome therapeutic quality.

MSC‐ex further contribute to angiogenesis by enhancing the production of VEGF [95, 97, 151, 152]. The most marked increase in VEGF levels was observed in rat cardiomyocytes exposed to adipose tissue (AT) MSC‐ex, compared to MSC‐ex derived from the BM or umbilical cord [153]. Exosomes have been shown to activate protein kinase A (PKA) in target cells, which subsequently enhances downstream transcription of VEGF and other pro‐angiogenic genes (*angiopontin‐1* and *receptor tyrosine kinase FLK‐1*) while suppressing anti‐angiogenic ones (*vasohibin‐1*) [109]. In addition, numerous pro‐angiogenic and pro‐migratory proteins have themselves been detected in MSC‐ex, including VEGF itself, cell adhesion proteins, and the cytokines transforming growth factor β (TGFβ) and interleukin‐8 (IL8) [154]. Therefore, MSC‐ex effectively orchestrate pro‐angiogenic effects through multifaceted mechanisms involving direct molecular signalling and delivery of various pro‐angiogenic factors.

Collectively, these findings underscore the promising therapeutic potential of MSC‐derived exosomes in myocardial regeneration, highlighting the significance of miRNA cargo, donor age, and MSC source in modulating their angiogenic efficacy. Future research into optimising exosome composition and sourcing could enhance their clinical application in myocardial repair.

#### 4.2.3 MSC‐Ex and Post‐Reperfusion Repair

Like CPC‐ex, MSC‐ex also demonstrate potential for post‐reperfusion repair. RI often results in counterproductive oxidative damage and inflammation. The subsequent increase in ROS activates reperfusion‐associated apoptotic pathways, preventing adequate restoration of the myocardium. Given its ability to induce ROS, H_2_O_2_ has been used in in vitro models to mimic the oxidative and pro‐apoptotic microenvironment associated with RI [98, 155]. In this context, murine AT‐MSC‐ex robustly reduced H_2_O_2_‐induced apoptosis in mouse cardiomyocytes by more than 50% [155]. BM‐MSCs were also effective in alleviating reperfusion‐associated apoptosis in rat H9c2 cardiomyocytes [98]. Interestingly, hypoxic culture media conditions enhanced secretion of BM‐MSC‐ex and stimulated their uptake by cardiomyocytes. In fact, treatment of H9c2 cells with these hypoxic exosomes for 12 h prior to H_2_O_2_ exposure significantly restored cell viability [98]. This exosome‐mediated anti‐apoptotic effect is partially due to activation of the AMPK/mTOR and Akt/mTOR pathways that eventually evoke autophagy‐related proteins like LC3B. Moderate autophagy during reperfusion imparts cardioprotective effects due to the elimination of otherwise harmful protein aggregates and destroyed organelles [156]. These observations are also reproduced using rat models of reperfusion injury post‐infarction [Table 1]. Injection of BM‐MSC‐ex into the peri‐infarct region 5 min prior to reperfusion resulted in increased LC3B expression, reduced apoptosis, diminished infarct region, and improved cardiac function. These findings underscore the therapeutic potential of MSC‐derived exosomes in mitigating reperfusion‐induced myocardial damage.

MSC‐ex exert these cardioprotective effects primarily through their rich miRNA cargo, which intricately orchestrates the regulation of cardiomyocyte survival, oxidative stress, and inflammation during reperfusion injury—working synergistically to enhance myocardial viability and promote tissue recovery. One of the most crucial reparative roles of MSC‐ex is their suppression of apoptosis, a major consequence of reperfusion injury. Importantly, this anti‐apoptotic effect is mediated by miR‐486‐5p, which targets and suppresses PTEN, a negative regulator of the PI3K/AKT pathway [157]. By inhibiting PTEN, miR‐486‐5p allows for sustained activation of Akt, promoting cell survival. Similarly, MSC‐ex carry several other anti‐apoptotic miRs including miR‐19a, −21, −22, −24, −125b, −144 [95], which collectively suppress the expression of apoptotic genes and activate pro‐survival pathways, most notably the PI3K/Akt/mTOR axis [95]. Additionally, miR‐143‐3p suppresses CHK2, a checkpoint kinase involved in DNA damage‐induced apoptosis, further reducing programmed cell death in stressed cardiomyocytes [158]. Through these miRNA‐regulated mechanisms, MSC‐ex significantly reduce cardiomyocyte loss during reperfusion and enhance myocardial preservation.

Oxidative stress and inflammation are two central and interconnected features of myocardial I/R injury, both of which contribute to cardiomyocyte death and impaired tissue recovery [110]. The abrupt generation of ROS during reoxygenation leads to cellular damage and apoptosis. BM‐MSC‐ex help restore redox balance by downregulating pro‐oxidative genes. One key mechanism involves miR‐183‐5p, which inhibits the expression of FOXO1, a transcription factor whose hyperactivation exacerbates oxidative stress and induces cardiomyocyte apoptosis by upregulating inducible nitric oxide synthase [159]. Nonetheless, miR‐150‐5p reduces the expression of thioredoxin‐interacting protein (TXNIP), a redox regulator that amplifies oxidative injury and inflammation when upregulated [160]. By downregulating TXNIP, not only does miR‐150‐5p exert antioxidant effects, but it also dampens inflammatory signalling by preventing TXNIP's activation of the NLRP3 inflammasome, a central driver of post‐I/R inflammation and fibrosis [161]. Taken together, BM‐MSC‐ex mitigate oxidative damage, suppress pathological inflammation, and preserve the functional viability of reperfused myocardial tissue, supporting a more regenerative post‐infarction environment.

Building on these findings from BM‐MSC‐ex, evidence has shown that MSC‐ex derived from human embryonic stem cells (HE‐MSC‐ex) also offers therapeutic promise in reperfusion therapy. Intravenous administration of HE‐MSC‐ex prior to reperfusion in mice models of induced MI resulted in a 50% reduction in infarct size within 24 h after reperfusion [97]. This cardioprotective effect was contingent upon exosome dosage, structural integrity, and direct uptake by cardiomyocytes. Notably, improvements in LVEF and overall cardiac function were observed as early as 1 day post‐reperfusion and were sustained throughout a 28‐day monitoring period [97], underscoring the therapeutic potential of HE‐MSC‐ex in I/R injury.

Mechanistically, these therapeutic effects appear to be mediated by several mechanisms including metabolic and antioxidant restoration along with the activation of pro‐survival signalling pathways. During I/R injury, mitochondrial dysfunction depletes ATP and NADH levels, and the widespread oxidative stress exhausts cellular antioxidants in the infarcted myocardium, contributing to further injury [162, 163]. Interestingly, HE‐MSC‐ex were shown to replete ATP and NADH levels, which was supported by proteomic analysis, revealing that HE‐MSC‐ex contain approximately 800 proteins primarily involved in glycolytic and oxidative pathways [97]. Moreover, these exosomes contained antioxidant enzymes such as peroxiredoxins and glutathione S‐transferases, which supplemented depleted cellular antioxidants and reduced oxidative stress within 1 h in exosome‐treated mice [97]. Adding to their therapeutic profile, these exosomes express enzymatically active membrane‐bound CD73, which catalyses the conversion of extracellular AMP to adenosine [164]. This adenosine subsequently activates the PI3K/Akt signalling pathway, which promotes cell survival and limits infarct size, as demonstrated in I/R mice models [165, 166]. Taken together, the observed therapeutic effects of HE‐MSC‐ex, similar to BM‐MSC‐ex, reinforce the notion that the secretion of reparative exosomes may be a generalizable and intrinsic property of mesenchymal stem cells, independent of their tissue source [97]. In fact, HE‐MSC‐ex may possess an added advantage over BM‐MSC‐ex. Unlike many prior studies that relied on hypoxic preconditioning to enhance BM‐MSC‐ex efficacy, the HE‐MSC‐ex used in the aforementioned studies were produced under normoxic conditions, yet still demonstrated high therapeutic efficacy. This feature is a key translational advantage, suggesting that HE‐MSC‐ex can mediate therapeutic effects without the need for hypoxic preconditioning, potentially simplifying their production for clinical application.

Ultimately, the presented evidence highlights the robust therapeutic potential of MSC‐derived exosomes in myocardial repair. Through their anti‐apoptotic effects, pro‐angiogenic signaling, and capacity to mitigate reperfusion‐induced damage, MSC‐ex offer a comprehensive approach to preserving and restoring cardiac function following ischaemic injury. Their molecular versatility, tissue compatibility, and consistent efficacy across different MSC sources underscore their promise as a next‐generation, cell‐free regenerative therapy for cardiovascular disease.

## 5 The Role of Induced Pluripotent Stem Cell‐Derived Exosomes (iPSC‐Ex) in Myocardial Repair

### 5.1 Therapeutic Potential of Induced Pluripotent Stem Cells (iPSCs) in Myocardial Repair

IPSCs are another set of stem cells which have emerged as promising candidates for cardiovascular regenerative therapy. IPSCs can be generated by genetically reprogramming adult human somatic cells, such as dermal fibroblasts, into a pluripotent embryonic stem cell (ESC)‐like form [167]. Consequently, iPSCs become similar to ESCs in many aspects, including their morphology, gene expression, proliferation and pluripotency [15]. Importantly, given their pluripotent state, iPSCs can differentiate into any cell type, including cardiomyocytes, which makes them promising candidates for cardiac repair [19]. Notably, iPSCs can differentiate into cardiomyocytes when subjected to similar protocols and culture conditions as those used for ESCs, which facilitates clinical translation [168]. In fact, iPSC‐based therapies offer several advantages over ESCs. For instance, generating iPSCs from human somatic cells provides an unlimited source of patient‐specific cells, which allows for personalised treatment approaches, potentially reducing the risk of immunological rejection [60]. Additionally, this technology enables the use of pluripotent stem cells while mitigating the ethical issues associated with ESCs [127]. These features collectively position iPSCs as a versatile and ethically favourable alternative to ESCs, with strong potential for advancing personalised cardiac regenerative therapies.

In the context of MI, iPSCs have demonstrated promising cardioprotective effects, particularly by virtue of their ability to differentiate into iPSC‐derived cardiomyocytes (iPSC‐CMs), which improve cardiac function [169]. IPSC‐CMs are polymerised into a sheet that can be directly transplanted over the cardiac region that has undergone infarction [170]. Following MI, transplantation of these iPSC‐CMs onto the injured area restored contractility and improved myocardial repair. Mechanistically, it appears that iPSC‐CMs are capable of integrating electromechanically with the host's cardiac muscle cells, where they release angiogenic factors and cytokines that improve the viability of damaged cells [171]. Moreover, iPSC‐CMs improved cardiac function by enhancing cardiac oxygen consumption, suppressing apoptosis of ischaemic cardiomyocytes, reducing myocardial wall stress, and ameliorating left ventricular function [172]. Together, these observations support the cardioprotective effects of iPSC‐CMs post‐MI.

Beyond cardiomyocytes, iPSCs can differentiate into other cardiovascular cells, including endothelial cells (ECs) and epicardial cells (EPs), which also offer unique contributions to myocardial repair. iPSC‐derived ECs (iPSC‐ECs), identified by CD31 expression, were capable of surviving post‐implantation, where they promoted revascularization and tissue regeneration [173]. More importantly, these iPSC‐ECs significantly enhanced survival rates in a mouse MI model, highlighting their therapeutic potential in cardiac repair [173]. Similarly, iPSC‐derived EPs, when implanted into infarcted murine and porcine hearts, have been shown to reduce fibrosis, promote angiogenesis, and modulate immune responses by enhancing reparative macrophage polarisation [174]. These findings suggest that a combination of several iPSC‐derived cardiac cells may offer synergistic therapeutic benefits in the context of post‐MI myocardial repair.

#### 5.1.1 Limitations of iPSC Therapy and the Rise of iPSC‐Ex in Myocardial Repair

It is important to bear in mind that utilising iPSCs to treat MI is not without limitations that could restrict their therapeutic function. To avoid immune rejection, transplantation of autologous host‐derived iPSCs is usually performed [175]. However, this approach is expensive and time‐consuming since a large number of cells are required. Alternatively, an allogeneic transplantation could resolve this problem, but it carries the risk of immune rejection mediated by cytotoxic T cells, anti‐HLA antibodies and NK cells [169, 176]. Immunosuppressive treatments can mitigate this effect but come with their own drawbacks, such as increasing the host's vulnerability to infections [19]. Nonetheless, generating iPSCs involves the delivery of reprogramming factors via retroviral vectors, which raises concerns regarding genomic instability and tumorigenic potential [15], although in vivo studies report similar teratoma rates between iPSCs and ESCs [60].

Compounding these issues, iPSC‐CMs face functional limitations that restrict their therapeutic application. One major challenge is their immature phenotype: iPSC‐CMs more closely resemble fetal rather than adult cardiomyocytes, as highlighted by their round shape, disorganised sarcomeres, lack of T‐tubules and underdeveloped mitochondria [177, 178]. This immaturity results in poor excitation‐contraction coupling, reduced contractility, and limited metabolic capacity, as iPSC‐CMs rely predominantly on glycolysis instead of mitochondrial oxidative phosphorylation [179, 180]. Furthermore, transplanted iPSC‐CMs have been linked to ventricular arrhythmias in large animal models [171], a phenomenon termed ‘engraftment arrhythmias’. These arrhythmias are believed to stem from the automaticity of immature cardiomyocytes—their ability to spontaneously depolarize and generate action potentials [19]. Notably, innovative genetic approaches that suppress automaticity, such as silencing depolarization‐associated genes while enhancing hyperpolarizing currents, have shown promise in reducing this risk [181]. Despite improvements in purification techniques and differentiation protocols, the risk of tumorigenicity due to residual undifferentiated cells persists [182]. Hence, while iPSC therapy offers substantial regenerative promise, key barriers including arrhythmogenic risk, functional immaturity, tumorigenic potential and immunogenicity must be addressed before widespread clinical translation is feasible.

These limitations have driven growing interest in iPSC‐derived exosomes (iPSC‐ex), which offer a cell‐free alternative that retains the regenerative benefits of iPSC therapy while mitigating its associated risks. Some of the therapeutic effects of iPSCs are increasingly understood to be mediated through their paracrine activity—particularly via the release of exosomes enriched with functional microRNAs [183]. For instance, exosomes secreted from iPSC‐CMs carry abundant miRNAs such as miR‐30, miR‐100‐5p, miR‐21‐5p and miR‐10a‐5p, which are involved in cardioprotection, angiogenesis, and anti‐apoptotic signalling [19, 184]. Similarly, iPSC‐EC exosomes contain miR‐1‐3p, miR‐100‐5p and miR‐199b‐5p, which contribute to vascular regeneration and endothelial function [184, 185, 186]. These findings underscore the critical role of iPSC‐derived exosomes in mediating the regenerative benefits of iPSC therapy through targeted intercellular communication. More importantly, iPSC‐ex therapy circumvents many of the barriers associated with iPSCs. Unlike cell‐based therapies, iPSC‐ex significantly minimise the risk of teratoma formation because they are non‐replicative. Their acellular nature significantly lowers the risk of immune rejection and the potential need for immunosuppressive regimens. Moreover, since iPSC‐ex do not involve transplanting iPSC‐CMs into myocardial tissue, this essentially minimises the risk of engraftment arrhythmias [175]. In light of these advantages, iPSC‐ex represent a compelling alternative to direct iPSC transplantation. Not only do these exosomes preserve the core regenerative functions of their parent cells, but they also overcome critical barriers such as tumorigenicity, arrhythmogenicity and immune incompatibility.

### 5.2 Therapeutic Mechanisms of iPSC‐Derived Exosomes in Myocardial Repair

Among the least studied stem cell‐derived exosomes in myocardial repair are those derived from iPSCs. Nevertheless, iPSC‐ex have also demonstrated favourable outcomes, showing considerable promise as a cell‐free alternative to direct stem cell transplantation. The therapeutic actions of iPSC‐ex are primarily mediated through three core mechanisms: suppression of cardiomyocyte apoptosis, promotion of angiogenesis, and protection against reperfusion injury. The following subsections explore these mechanisms in detail, highlighting the molecular pathways involved and the regenerative potential of iPSC‐ex in the context of MI.

#### 5.2.1 IPSC‐Ex and Anti‐Apoptotic Activity

A key therapeutic mechanism of iPSC‐ex in myocardial repair is their ability to suppress cardiomyocyte apoptosis. These exosomes are rich in bioactive microRNAs that target distinct signalling pathways, collectively working to preserve cardiomyocyte viability and prevent infarct expansion. Notably, multiple miRNAs within iPSC‐ex act in concert to regulate intracellular calcium, inhibit apoptotic proteins, and activate pro‐survival signalling cascades.

One of the most critical miRNAs in this context is miR‐100‐5p, abundantly present in exosomes derived from iPSC‐endothelial cells (iPSC‐EC‐ex). This miRNA targets protein phosphatase 1β (PP‐1β), leading to enhanced phosphorylation of phospholamban (PLB) at Ser [16, 185]. The result is increased SERCA activity, which promotes efficient calcium reuptake into the sarcoplasmic reticulum, thereby improving excitation–contraction coupling and reducing calcium overload—a major contributor to cell death in ischaemic myocardium [187, 188]. By restoring calcium homeostasis, miR‐100‐5p enhances cardiomyocyte contractility and survival following oxygen–glucose deprivation (OGD), and synergizes with other miRNAs in iPSC‐ex to facilitate functional recovery after myocardial infarction [185].

Complementing this pathway, miR‐21 and miR‐210, both enriched in iPSC‐ex, play equally vital roles in cardiomyocyte protection [100]. miR‐21 inhibits PTEN, thereby activating the PI3K/Akt signalling pathway, which is well established for its anti‐apoptotic effects [189]. Activated Akt promotes downstream survival signalling and inhibits caspase activation, limiting programmed cell death in injured myocardium [190]. Meanwhile, miR‐210 decreases mitochondrial ROS production, further reducing cardiomyocyte cell death and supporting myocardial cell integrity under hypoxic conditions [191]. In vivo evidence supports these mechanistic findings. In mouse models of myocardial infarction, intra‐myocardial injection of iPSC‐exosomes reduced cardiomyocyte apoptosis by approximately 70% within 24 h of reperfusion. This was accompanied by a significant downregulation of caspase‐3, a central executor of apoptosis, and correlated with elevated levels of miR‐21 and miR‐210 within treated tissues [100]. Similar anti‐apoptotic effects have also been observed in large animal MI models treated with iPSC‐ex, underscoring the translational relevance of these findings [192].

Notably, compared to exosomes derived from cardiac fibroblasts (CF‐ex), iPSC‐ex contain significantly higher levels of miR‐21 and miR‐210. Importantly, their expression appears to be regulated by pluripotency‐associated transcription factors: Nanog and HIF‐1α [100]. miR‐21 is transcriptionally regulated by Nanog, a core pluripotency factor in embryonic and induced pluripotent stem cells. Silencing Nanog in stem cells results in a significant decrease in miR‐21 expression, indicating that miR‐21 is a downstream mediator of Nanog‐driven survival pathways [193]. On the other hand, miR‐210 is directly regulated by HIF‐1α, a transcription factor activated under hypoxic conditions [194]. HIF‐1α is upregulated in both the embryonic stem cell niche and the ischaemic myocardium, and its activation drives miR‐210 expression as part of the adaptive response to low oxygen [195, 196]. Hence, this transcriptional control ensures that miR‐210 is enriched in iPSC‐exosomes and delivered to injured cardiomyocytes during MI, which is precisely when its protective functions are most needed.

Taken together, the cooperative actions of miR‐100‐5p, miR‐21 and miR‐210 form a complex and synergistic regulatory network that targets multiple apoptotic and survival pathways. This coordinated molecular activity enables iPSC‐exosomes to effectively limit cardiomyocyte death, preserve myocardial function, and support tissue recovery after ischaemic injury. Their multifaceted anti‐apoptotic mechanisms reinforce the potential of iPSC‐ex as a robust, cell‐free therapy in cardiac regeneration.

#### 5.2.2 IPSC‐Ex and Pro‐Angiogenic Activity

A critical reparative function of iPSC‐ex lies in their ability to promote angiogenesis, an essential process for restoring perfusion and supporting tissue regeneration in the ischaemic myocardium. For example, mouse fibroblast‐derived iPSC‐ex are notably enriched with a range of pro‐angiogenic miRNAs and proteins, including miR‐19b, miR‐20a, miR‐126‐3p, miR‐130a‐3p, miR‐210‐3p, as well as vascular endothelial growth factor (VEGF), bone morphogenetic protein‐4 (BMP‐4), and platelet‐derived growth factor α (PDGFα) [99]. These components function collectively to activate angiogenic signalling pathways and promote endothelial cell proliferation, migration, and tube formation [99]. In vitro, murine cardiac endothelial cells (CECs) demonstrated rapid uptake of iPSC‐ex within 1 h, followed by robust capillary‐like tube formation within 7 h, confirming the strong angiogenic potency of iPSC‐ex [99].

Among the key regulators in this process is the miR‐17–92 cluster, which includes miR‐19a, miR‐19b and miR‐20a [197]. This cluster is transcriptionally upregulated by VEGF through the ERK/Elk‐1 signalling pathway, enhancing endothelial cell proliferation and angiogenic sprouting [198]. Mechanistically, the cluster promotes angiogenesis by targeting PTEN, a negative regulator of the PI3K/Akt pathway. By suppressing PTEN, the miR‐17–92 cluster facilitates VEGF‐mediated endothelial proliferation and vascular branching [198]. Additional miRNAs within iPSC‐exosomes, including miR‐126‐3p, miR‐130a‐3p and miR‐210‐3p, further enhance angiogenesis through various mechanisms [99]. miR‐126‐3p supports endothelial cell survival and angiogenic signalling by directly targeting and degrading SPRED1 and PIK3R2, both of which inhibit downstream VEGF signalling pathways such as ERK and PI3K/Akt [199]. Hence, miR‐126‐3p promotes VEGF signalling, thereby enhancing endothelial proliferation and migration, especially under ischaemic conditions. In parallel, miR‐130a‐3p reinforces the angiogenic phenotype of endothelial cells by suppressing the anti‐angiogenic transcription factors GAX and HOXA5, thereby lifting transcriptional repression of angiogenic programs [200]. Additionally, miR‐210‐3p plays a crucial role in hypoxia‐induced neovascularization. Under oxygen–glucose deprivation, miR‐210‐3p enhances endothelial progenitor cell (EPC) proliferation, migration, and tube formation by targeting and inhibiting repulsive guidance molecule A (RGMA), a negative regulator of angiogenesis [201]. Together, these miRNAs form an integrated regulatory network that activates angiogenic signalling cascades, modulates transcriptional repression, and adapts to hypoxic stress, amplifying the reparative capacity of iPSC‐exosomes in the ischaemic myocardium.

Ultimately, the pro‐angiogenic potential of iPSC‐derived exosomes is driven by a coordinated network of miRNAs and growth factors that target multiple molecular pathways involved in endothelial activation and vascular remodelling. Through the synergistic actions of the miR‐17–92 cluster, miR‐126‐3p, miR‐130a‐3p and miR‐210‐3p, iPSC‐exosomes not only enhance VEGF signalling and relieve anti‐angiogenic repression, but also adapt to ischaemic and hypoxic conditions. This multifaceted regulation positions iPSC‐ex as a potent therapeutic platform capable of promoting sustained neovascularization and functional recovery in ischaemic myocardial tissue.

#### 5.2.3 IPSC‐Ex and Post‐Reperfusion Repair

Another therapeutic application of iPSC‐ex lies in their ability to promote post‐reperfusion myocardial repair. In murine models of MI, intra‐myocardial injection of iPSC‐exosomes post‐reperfusion therapy led to significantly improved cardiac outcomes by day 35, including enhanced cardiac function and myocardial tissue repair [99]. Interestingly, while both iPSCs and iPSC‐ex promoted neovascularization in non‐ischaemic areas, only iPSC‐exosomes effectively stimulated angiogenesis in the infarct region, suggesting superior targeting and efficacy within ischaemic tissues. Additionally, a marked anti‐apoptotic effect was observed by day 35, particularly within cardiomyocytes [99], highlighting the post‐reperfusion reparative potential of exosome‐based therapy beyond the effects of cell transplantation alone.

One of the central challenges of reperfusion therapy is the paradoxical injury caused by the reintroduction of oxygen to previously ischaemic tissue. This sudden oxygen influx generates a burst of ROS, leading to oxidative stress, mitochondrial dysfunction, and activation of apoptotic pathways [110]. However, iPSC‐exosomes have been shown to effectively counteract these effects. Studies have shown that iPSC‐exosomes are efficiently internalised by cardiomyocytes in vitro and exert robust protective effects against oxidative stress. For example, in H9C2 cardiomyoblasts exposed to H_2_O_2_‐induced injury, iPSC‐ex treatment significantly suppressed caspase‐3/7 activation, indicating reduced apoptotic signalling [100, 202]. Similarly, in vivo transplantation of iPSC‐ex into ischaemic myocardium led to decreased active caspase‐3 protein expression and preserved cardiomyocyte viability, further validating their anti‐apoptotic role during the vulnerable reperfusion phase [100].

At the molecular level, the therapeutic efficacy of iPSC‐exosomes during reperfusion is largely driven by their delivery of cardioprotective microRNAs, especially miR‐21 and miR‐210, into stressed cardiomyocytes [100]. These miRNAs work through complementary mechanisms to combat oxidative damage and apoptosis. MiR‐21 activates the PI3K/Akt signalling pathway via PTEN inhibition, supporting cell survival under ischaemic conditions [189]. Meanwhile, miR‐210 mitigates oxidative stress by suppressing mitochondrial ROS production [191]. Overexpression of miR‐210 has been shown to reduce ROS accumulation and prevent cell death following hypoxia‐reoxygenation, while its downregulation leads to increased oxidative injury [196]. These findings underscore the role of iPSC‐exosomes as dynamic regulators of redox homeostasis and resistance to apoptosis during myocardial reperfusion.

Taken together, iPSC‐ex offer a highly targeted approach to mitigating the detrimental effects of reperfusion injury. By delivering key microRNAs such as miR‐21 and miR‐210, they restore redox balance, suppress apoptosis, and promote tissue regeneration within the infarcted myocardium. Their ability to modulate both cellular survival pathways and oxidative stress responses positions iPSC‐ex as a promising therapeutic strategy for enhancing myocardial recovery and long‐term functional outcomes following ischaemia–reperfusion. However, to better characterise the effect of iPSC‐ex in myocardial repair, further studies are warranted to elucidate their full therapeutic potential and optimise their clinical application.

## 6 Exosome Uptake by Different Cardiac Cells

Understanding the cellular uptake dynamics of stem cell‐derived exosomes is critical to elucidating their targeted therapeutic actions in cardiac repair, as different exosome types exhibit varying uptake levels and effects on cardiomyocytes, endothelial cells, and fibroblasts. For instance, CPC‐ex are effectively taken up by cardiomyocytes, leading to a time and dose‐dependent increase in anti‐apoptotic miRNAs, such as miR‐210 and miR‐132 [80, 203]. Although direct evidence of CPC‐ex uptake by endothelial cells remains limited, these exosomes have been shown to promote endothelial cell migration and angiogenesis. This effect may be mediated in part through interactions involving extracellular matrix metalloproteinase inducer (EMMPRIN) and matrix metalloproteinases (MMPs), as well as the delivery of exosomal pro‐angiogenic microRNAs, as discussed in Section 3.3.2 [80, 204, 205]. Similarly, there is little evidence demonstrating CPC‐ex uptake by cardiac fibroblasts. Nonetheless, BM‐MSC‐ex and HE‐MSC‐ex are taken up by cardiomyocytes in a dose‐dependent manner, with uptake notably enhanced under hypoxic conditions [92]. These exosomes transfer regulatory microRNAs that promote endothelial activation and angiogenesis [91, 92, 93, 94], which has been shown to decrease cardiac fibrosis after ischaemia–reperfusion [175]. Similarly, cardiomyocytes are capable of endocytosing exosomes derived from iPSC‐derived cardiomyocytes, leading to improved cardiac function in murine models [206]. In addition, iPSC‐ex are taken up by cardiac endothelial cells, leading to their proliferation and migration, which mediates angiogenesis [99]. These observations underscore the importance of exosome origin in dictating cellular uptake and functional outcomes, suggesting that optimising exosome‐cell targeting could significantly enhance the precision and efficacy of myocardial repair strategies.

## 7 Comparative Therapeutic Profile of Stem Cell‐Derived Exosomes

Exosomes derived from different stem cell sources, namely CPCs, MSCs, and iPSCs, demonstrate overlapping yet distinct therapeutic profiles in the setting of MI. Although they share several mechanisms such as anti‐apoptotic signalling, angiogenesis promotion, and post‐reperfusion repair, comparative evaluation reveals key differences in efficacy, safety, and translational feasibility [Table 2].

**TABLE 2:** Comparative therapeutic profile of exosomes from CPCs, MSCs and iPSCs in myocardial repair.

Aspect of comparison	CPC‐derived exosomes	MSC‐derived exosomes	iPSC‐derived exosomes
Key exosomal cargo			
Anti‐apoptotic	miR‐21, miR‐210	miR‐25‐3p, miR‐21, miR‐24, miR‐19a, miR‐143‐3p, miR‐210, ITCH, MFGE8	miR‐100‐5p, miR‐21, miR‐210, miR‐30, miR‐10a‐5p
Pro‐angiogenic	miR‐132, miR‐210, miR‐322, miR‐20, VEGF, MMP‐9, EMMPRIN	miR‐205, miR‐543, miR‐486‐5p, miR‐132, miR‐221‐3p, miR‐21, miR‐1246, VEGF, TGF‐β, IL‐8	miR‐19a/b, miR‐20a, miR‐126‐3p, miR‐130a‐3p, miR‐210‐3p, miR‐1‐3p, miR‐100‐5p, miR‐199‐5p, VEGF, BMP‐4, PDGFα
Post‐reperfusion repair	miR‐451, miR‐146a, miR‐29c, miR‐27a, miR‐22, miR‐24	miR‐486‐5p, miR‐19a, miR‐21, miR‐22, miR‐24, miR‐150‐5p, miR‐183‐5p, miR‐125b, miR‐143‐3p, miR‐144, peroxiredoxins, glutathione S‐transferases, glycolytic enzymes	miR‐21, miR‐210
Therapeutic effects			
Cardiac function	Improved systolic/diastolic function; ↑↑ LVEF with CPC‐ex^CXCR4^	↑ LVEF (day 7–28); HE‐MSC‐ex improved EF within 1 day	Improved cardiac function by day 35 post‐reperfusion
Infarct size	↓ Apoptosis by 53% post‐reperfusion	↓ Infarct size with BM‐MSC‐ex and HE‐MSC‐ex	↓ Infarct size by day 35 post‐reperfusion
Cardiac remodelling	↓ Fibrosis via miR‐146a/SMAD4	Apoptotic clearance via MFGE8	Anti‐fibrotic effect via miR‐210, miR‐126‐3p
Cardiomyocyte survival	↓ Caspase 3/7; ↑ myocardial viability via miR‐21, miR‐210	↓ Apoptosis via miR‐25‐3p, miR‐21, miR‐24, miR‐19a; ↑ myocardial viability via ITCH	~70% ↓ apoptosis (24 h); ↓ caspase‐3; ↑ cardiomyocyte survival via miR‐100‐5p, miR‐21, miR‐210
Angiogenesis	↑ Tube formation; ↑ vessel density via VEGF, MMP‐9, EMMPRIN, miR‐132, −210	↑ Tube formation via angiogenic miRs: miR‐205, −543, −486‐5p, −132; ↑VEGF	↑ Tube formation (7 h) via angiogenic miRs: miR‐19b, −20a, −126‐3p, −130a‐3p, −210‐3p; ↑VEGF
Mitigating I/R injury	miR‐451 (COX‐2/PGE2 survival pathway); miR‐146a (↓ inflammation/ROS)	↑ LC3B, ↓ apoptosis, ↑ ATP/NADH, antioxidant enzymes; miR‐183‐5p, −150‐5p	↓ Caspase‐3/7; ↓ ROS via miR‐21 (PI3K/Akt), miR‐210 (antioxidant); improved recovery by day 35

Safety profile	Low risk of immunogenicity, arrhythmogenicity and tumorigenicity		
Preparation difficulty	Moderate	Low	High
	Requires invasive procedures to obtain cardiac tissue source and may need specific isolation protocols due to the rarity of CPCs in tissues	Many MSC sources with well‐established methods of cell culture and isolation techniques	Technically demanding with complex iPSC reprogramming protocols
Experimental stage	Preclinical trials	Phase 1/2 clinical trials	Preclinical trials

In terms of therapeutic efficacy, meta‐analytic findings from 37 preclinical animal studies show that MSC‐ex exhibit the most robust improvements in LVEF, with a mean difference (MD) of +11.72% [95% CI: 9.17, 14.48], followed by CPC‐ex (MD: +8.78% [95% CI: 6.11, 11.45]) [207]. However, when secondary outcomes such as end‐systolic volume (ESV), end‐diastolic volume (EDV), and myocardial wall thickness are considered, CPC‐ex tend to be superior. For instance, CPC‐derived exosomes were associated with the largest reductions in ESV (−0.45 μL) and EDV (−0.19 μL), along with the highest increase in wall thickness (+0.29 mm), suggesting enhanced prevention of adverse cardiac remodelling [207]. In contrast, MSC‐ex showed the most pronounced reductions in infarct size (−13.59%), reflecting strong early infarct containment benefits [207]. iPSC‐derived exosomes also showed favourable effects, but with relatively limited quantitative data in large animal models. Nonetheless, preclinical studies demonstrate a ~70% reduction in cardiomyocyte apoptosis within 24 h of reperfusion and significant functional improvements which lasted up to 35 days post‐MI [99].

Mechanistically, all three exosome types exert anti‐apoptotic, pro‐angiogenic, and post‐reperfusion reparative effects via their unique miRNA and protein cargo. CPC‐ex are rich in miR‐21, miR‐210, miR‐132, miR‐451 and miR‐146a, which target key apoptotic, angiogenic, and inflammatory pathways [80]. MSC‐ex carry a broader panel of anti‐apoptotic (miR‐25‐3p, miR‐21, miR‐24) and pro‐angiogenic miRNAs (miR‐205, miR‐132, miR‐1246), along with immunomodulatory proteins like MFGE8 [37]. Meanwhile, iPSC‐exosomes deliver pluripotency‐linked miRNAs such as miR‐100‐5p, miR‐21, miR‐210 and miR‐126‐3p that modulate intracellular calcium cycling, oxidative stress and vascular regeneration [99, 185].

In terms of safety, exosomes offer a major advantage over direct stem cell transplantation by minimising risks such as teratoma formation, engraftment arrhythmias, and immune rejection [78, 208]. Indeed, CPC‐ex, MSC‐ex and iPSC‐ex have all demonstrated favourable safety profiles in experimental trials across a range of diseases, including MI [208, 209, 210]. However, it is important to note that MSC‐ex have been the most extensively evaluated, with relatively less safety data available for CPC‐ex and iPSC‐ex. In the context of using exosomes for myocardial repair, the primary safety concern lies in the methods of stem cell extraction and exosome delivery to the injured myocardium, particularly if invasive procedures are involved. However, when it comes to the exosomes themselves, preliminary results from ongoing clinical trials show promising safety profiles [208]. Yet, further trials are warranted to assess the safety of exosomes in the long term and in larger, more diverse populations.

In terms of production and scalability, MSC‐ex are the most accessible. MSCs can be isolated from a wide range of adult tissues and expanded using standard protocols, making their exosome products the most amenable to upscaling and clinical‐grade manufacturing [211]. CPC‐derived ex, although potent, are less scalable due to the rarity of CPCs in adult tissues and the need for invasive harvest procedures [83]. Isolation of pure CPC populations also remains technically demanding [212]. iPSC‐ex, while potentially unlimited in supply, require complex reprogramming protocols to produce them from somatic sources, which complicates their clinical translation [213].

Taken together, MSC‐ex show the strongest improvements in cardiac function and infarct size, CPC‐ex excel in preventing adverse cardiac remodelling, and iPSC‐exosomes offer promising long‐term reparative effects. All three types of exosomes exhibit promising safety profiles. However, CPC‐ex and iPSC‐ex face practical limitations in isolation and production, respectively. Hence, these findings suggest that the optimal choice of exosome source may depend on therapeutic priorities and should be guided by further head‐to‐head studies in clinically relevant models.

## 8 From Bench to Bedside: Challenges in the Clinical Application of Exosomes

While exosome therapy holds significant promise for regenerative medicine and myocardial repair, several key challenges continue to hinder its widespread adoption in clinical settings. These range from safety concerns and variability in biological content to difficulties in standardisation and delivery optimisation. One major concern pertains to the fact that natural exosomes are highly heterogeneous and carry a diverse array of biomolecules, such as miRNAs, proteins, and lipids, whose functions are not yet fully understood. For instance, a single miRNA can regulate numerous genes, raising concerns over potential off‐target effects [214]. Environmental changes can also alter exosome content, making it difficult to standardise treatments [215]. As a result, implementing rigorous quality control protocols across all stages of production, from cell sourcing and culture conditions to isolation, characterisation, and storage, is critical to ensure the therapeutic consistency and safety of exosomes.

Another barrier is the variability in exosome yield and composition across different biological fluids and patient populations. Factors such as age, sex, disease state, and lifestyle significantly affect the quality and quantity of exosomes [216]. This variability necessitates customised and optimised protocols for each specimen type. Furthermore, degradation during the isolation process can compromise therapeutic value. Various isolation methods such as ultracentrifugation, microfluidics, and size exclusion chromatography have been developed, but none offer a universal standard. Each method has trade‐offs between purity, yield, scalability and preservation of exosomal integrity [217]. Storage and preservation add another layer of complexity: repeated freeze–thaw cycles can degrade the vesicles or reduce their functional potency, and common preservatives used such as EDTA or heparin can alter exosome stability [218, 219]. After isolation, characterisation of exosomes is essential to determine therapeutic dosing, but this also lacks standardisation. Techniques such as nanoparticle tracking analysis, flow cytometry, transmission electron microscopy, ELISA for CD63 (exosome surface marker), and polycarbonate filtration all produce variable results, limiting reproducibility and regulatory acceptance [220, 221]. For clinical translation, harmonised protocols for identification, quantification, and functional validation of exosomes are urgently needed.

Beyond quality control, dosage and administration optimization also pose significant barriers. In animal models, systemic administration routes such as intravenous or intraperitoneal injection often result in rapid clearance by the mononuclear phagocyte system in the liver and spleen [222]. This short plasma half‐life leads to poor retention in target sites and necessitates repeated high doses for therapeutic effect. To combat this, researchers are exploring modifications such as incorporating camouflage proteins into exosome membranes to enhance immune evasion and prolong circulation time [223]. Enhancing these delivery systems, alongside in vivo tracking to further elucidate exosome biodistribution, will be essential to overcoming these barriers and facilitating the clinical application of exosomes.

## 9 Future Prospects and Avenues for Improvement

### 9.1 Optimising Exosome Delivery: Emerging Routes of Administration

Effective delivery methods are essential to unlock the full regenerative potential of exosome‐based therapies in cardiac repair. Traditional administration routes such as intravenous or intramyocardial injections have been widely used; however, they face significant challenges including poor retention of exosomes at target sites, inefficient homing to damaged tissue, and short biological half‐lives [21]. These limitations often necessitate multiple repeated injections to achieve significant therapeutic effects, which can be burdensome for patients and may reduce treatment efficacy [24]. Cardiac patches have emerged as a promising solution, as they can retain exosomes locally and provide sustained release, thereby improving therapeutic outcomes [21]. For instance, exosomes derived from human umbilical cord mesenchymal stem cells loaded into fibrin‐based patches mimic the extracellular matrix and have demonstrated improved retention in infarcted hearts, reduced fibrosis, and enhanced cardiac function in preclinical studies [224]. Despite these advantages, the invasive nature of cardiac patch implantation restricts its practicality and wider clinical adoption.

To address these concerns, minimally invasive and non‐invasive delivery strategies that maintain therapeutic efficacy are currently under investigation. Injectable hydrogels, which can be applied directly onto ischaemic myocardium, offer controlled and biodegradable release of exosomes while minimizing surgical intervention [225]. A notable innovation is the use of hydrogel sprays such as the ‘EXOS’ system composed of thrombin and MSC‐derived exosomes in a fibrinogen solution. Such hydrogels solidify upon contact with target tissue, stabilising exosome cargo and improving targeted delivery [226]. Additionally, aerosolised delivery of exosomes via nebulization represents a novel non‐invasive approach with promising preclinical outcomes [227]. The SCENT (stem cell‐derived exosome nebulization therapy) technique delivers lung spheroid stem cell exosomes through inhalation. Importantly, this delivery strategy has proven to improve ventricular function, reduce fibrosis, and enhance cardiomyocyte proliferation in animal models, without eliciting adverse systemic effects [227]. These emerging delivery routes are transforming exosome therapy by enhancing retention, targeting, and therapeutic efficacy, while reducing procedural invasiveness.

### 9.2 From Small Packages to Smaller Solutions: Enhancing Exosome Therapy With Nanoparticles

Combining nanoparticles (NPs) with exosomes presents a promising strategy to enhance targeted therapeutic delivery, especially in the management of myocardial infarction [228]. One innovative approach involves the use of core‐shell magnetic nanoparticles conjugated with two antibodies: one targeting CD63, an exosomal surface marker, and the other recognizing myosin light chain (MLC), a marker of damaged cardiomyocytes [229]. This dual‐targeting design enables the NPs to serve as a vesicle shuttle system, capturing exosomes and then selectively delivering them to the damaged cardiac tissue. Upon reaching the site of injury, the exosomes are released, carrying out their therapeutic effects, including angiogenesis, reduction in infarct size, and improved left ventricular ejection fraction, as demonstrated in animal models [229].

Another intriguing feature of nanoparticle‐exosome systems lies within their potential to reduce the harmful accumulation of pathological exosomes. Evidence has shown that exosomes containing pro‐inflammatory and pro‐coagulation factors may contribute to thrombosis and exacerbate injury in myocardial infarctions [230]. By conjugating NPs with antibodies specific to these harmful exosome subtypes, it is possible to neutralise or remove them from circulation before they reach the infarcted myocardium [228]. Hence, using NPs to deliver therapeutic exosomes while intercepting pathological ones offers a promising potential to maximise the therapeutic efficacy of exosome‐based therapy in myocardial repair. However, optimization of specific target antigen selection and exosome release kinetics remains a field of ongoing research. Nonetheless, a novel strategy where exosome nanocomplexes were encapsulated with low molecular weight heparin has been shown to overcome microvascular obstruction in the infarcted myocardium [231]. Not only did this improve exosome delivery to the infarcted area, but it also facilitated the uptake of therapeutic miRNAs by cardiomyocytes leading to decreased apoptosis, attenuated myocardial fibrosis, and enhanced cardiac repair [231]. Ultimately, the integration of nanotechnology in exosome‐based therapy offers a promising potential for improving outcomes in acute myocardial infarction.

### 9.3 Enhancing Exosome Tropism: Chemical and Genetic Engineering Strategies

While promising, exosome‐based therapy requires further investigation in its efficient and safe delivery into the injured heart muscle. Numerous factors, such as chemical or genetic treatment, may impact exosome functionality and tropism towards cardiac cells. For example, exosomes derived from sulforaphane‐treated fibroblasts have shown higher affinity and tropism towards cardiomyocytes, leading to decreased hypertrophy and apoptosis [232]. This modification may have a similar impact on exosomes derived from other cells, such as CPCs or MSCs, further improving their therapeutic effect. Moreover, genetic modification may play a pivotal role in intensifying the efficacy of exosome‐based therapy. This was highlighted in a study where exosomes were engineered to express a cardiomyocyte‐specific binding peptide (CMP) bound to a naturally present protein, Lamp2b, on exosome membranes. These exosomes showed preferential endocytosis compared to non‐modified exosomes [233]. This method not only improves the effectiveness of exosomes but may also help avoid off‐target effects. Another promising approach involves leveraging the ability of stromal cell‐derived factor‐1 alpha (SDF‐1α) to bind to CXCR4 receptors. Following MI, the infarcted myocardium exhibits rapid upregulation of SDF‐1α, primarily driven by hypoxia‐inducible factor 1 (HIF‐1) [234, 235]. This creates a potent chemoattractant gradient that promotes recruitment of CXCR4‐expressing leukocytes and stem cells to the injured tissue to promote cardiac repair [236, 237]. In light of this endogenous mechanism, CPC‐derived exosomes engineered to overexpress CXCR4 (CPC‐ex^CXCR4^) were hypothesized to achieve superior homing to ischaemic myocardium, thereby increasing therapeutic cargo delivery to target cardiomyocytes. Indeed, in vivo systemic administration of CPC‐ex^CXCR4^ post‐reperfusion resulted in marked improvements in left ventricular systolic and diastolic function, as well as a reduction in infarct size, compared to control CPC‐ex [238] [Table 2]. These benefits were attributable to enhanced CPC‐ex^CXCR4^ homing to the infarct border zone, thereby increasing local bioavailability and uptake by cardiomyocytes. Importantly, this finding is clinically relevant, as systemic delivery offers a minimally invasive route that can be employed immediately following reperfusion therapy in acute MI settings, yet it is limited by poor cardiac homing, which can be improved with such engineered CPC‐derived exosomes. Collectively, these findings underscore the transformative potential of chemical and genetic engineering strategies to enhance the cardiac tropism, efficacy, and precision of exosome‐based therapies, paving the way for more targeted and clinically translatable interventions in myocardial repair.

### 9.4 Drug–Exosome Interactions: Therapeutic Implications

Other factors to consider include medications or drugs that may play a role in enhancing or diminishing exosome function in the ischaemic heart. On one hand, doxorubicin, a drug used for cancer treatment, has long been shown to have cardiotoxic effects. In fact, it has been shown to impact extracellular exosomes by altering their molecular composition, especially miRNAs, and by reducing their expression in stem cells and fibroblasts [239]. Amiloride has also been shown to interfere with multivesicular bodies formation, therefore inhibiting exosome biogenesis [240]. On the other hand, other drugs can be used to enhance exosome function. For example, ticagrelor has been shown to carry such a function by enhancing their release from CPCs and cardiomyocytes [241, 242]. Additionally, atorvastatin and carvedilol induce the release of exosomes that have cardioprotective and atherosclerotic functions, respectively [243, 244]. These results highlight the impact of drugs, whether taken for heart disease or for other reasons, on the efficacy of exosomes in cardioprotection. While being mindful of patients' drug intake and their possible known or unknown interaction with exosome therapy might be important, equally so, considering some medications as a non‐invasive approach to enhance naturally expressed exosome should be considered.

### 9.5 Age‐ and Sex‐Dependent Variability in Exosome‐Based Cardiac Repair

Aside from the above‐mentioned elements, two important factors that may come into play in the feasibility and efficacy of exosome‐based cardiac repair are age and sex. In fact, female‐derived exosomes from endometrial mesenchymal stem cells have been shown to have more cardioprotective properties compared to bone marrow or adipose‐derived stem cells. They were found to be richer in miR‐21, which has a direct effect on the PTEN‐Akt survival pathway, giving it an anti‐apoptotic and pro‐angiogenic function [245]. Moreover, oestrogen may also play a role in cardioprotection. In oestrogen receptor beta knockout mice, there was a decrease in the activation of the PI3K‐Akt signalling pathway, leading to worse post‐ischaemia myocardial function [246]. As for age, it plays an important role in the efficacy of stem cell‐derived exosome‐based therapy. When addressing the age factor, we differentiate between donor cell age and recipient age. Aged MSC‐derived exosomes were found to have lower levels of miRs, such as miR‐29, miR‐146a, and miR‐21, attenuating their ability to carry out cardiac repair efficiently [150, 247]. Interestingly, exosomes from aged hearts, particularly from aged male donors, exhibited a pro‐fibrotic effect on cardiac fibroblasts. In contrast, exosomes from younger cohorts, including females and young males, were found to have anti‐fibrotic effects [248]. This suggests that exosome behaviour changes with age, with aged exosomes contributing to a pro‐inflammatory and pro‐fibrotic environment in the myocardium. These findings highlight the importance of considering donor age when utilising exosome‐based therapies for cardiac repair, as the therapeutic potential of exosomes from older individuals may be less favourable due to these pro‐fibrotic effects. The differential impact of exosomes from young and aged hearts on cardiac fibroblasts points to the need for further research to better understand how exosome content and bioactivity are influenced by age and to optimise exosome therapies based on the patient's age and sex for more effective treatment outcomes.

### 9.6 Enhancing Stem Cell Therapy With Exosomes: A Synergistic Approach to Cardiac Repair

Stem cell‐based therapy has emerged as a promising strategy for cardiac regeneration, particularly following acute myocardial infarction. However, a major barrier limiting its clinical efficacy remains the low survival, poor engraftment, and limited retention of transplanted stem cells in the hostile ischaemic microenvironment [21]. These limitations have prompted increasing interest in strategies that can improve cell viability and therapeutic efficacy—among which the use of stem cell‐derived exosomes has gained considerable attention. Exosomes not only mediate critical intercellular signalling but also possess the capacity to modify the infarcted microenvironment, rendering it more conducive to stem cell survival and function [249].

Recent studies demonstrate that MSC‐ex can be strategically used to precondition the infarcted myocardium prior to stem cell transplantation. Injecting exosomes into the ischaemic heart as early as 30 min post‐infarction has been shown to modulate the local environment by attenuating inflammatory responses and enhancing SDF‐1 expression, which plays a pivotal role in recruiting stem cells [250]. Notably, early MSC‐ex delivery followed by MSC transplantation, particularly around day 3 post‐MI, yields superior cardiac outcomes compared to either treatment alone. This timing takes advantage of the peak in SDF‐1 expression while avoiding the early pro‐inflammatory window following MI, thereby creating a more permissive milieu for stem cell engraftment [250]. Importantly, this strategy led to significant results both in vitro and in vivo, including improved MSC retention, enhanced angiogenesis, reduced apoptosis, and restoration of cardiac function [250]. Similarly, sequential transplantation of exosomes and pretreated MSCs significantly improved cardiac repair compared to AMI rats treated with exosomes alone [251]. Mechanistically, intramyocardial delivery of exosomes 30 min post‐infarction effectively modulated the ischaemic milieu by reducing pro‐inflammatory cytokines such as TNF‐α and IL‐6, upregulating SDF‐1 expression, and enhancing the survival of subsequently transplanted MSCs [251]. Furthermore, dual pretreatment of MSCs with hypoxia and the pharmacological agent Tongxinluo (TXL) synergistically elevated CXCR4 expression, thereby amplifying their chemotactic response to SDF‐1. This optimised priming strategy enhanced MSC recruitment to the infarct border zone, promoted angiogenesis, reduced myocardial fibrosis, and led to superior long‐term functional recovery [251]. These findings offer compelling insight into the potential of exosome‐based combinatorial strategies to overcome existing limitations in stem cell‐mediated cardiac repair.

In addition to modifying the extracellular environment, exosomes directly enhance the intrinsic properties of stem cells. For instance, preconditioning CPCs with MSC‐ex boosts their proliferative and migratory capacity, promotes angiogenic signalling, and reduces fibrotic remodelling post‐MI [252]. This effect is largely mediated through the transfer of functional microRNAs that reprogram target cells. Notably, microarray analysis revealed distinct shifts in miRNA expression in CPCs after exosome treatment, including the downregulation of miRNAs that suppress VEGF signalling and angiogenesis. Interestingly, these changes corresponded with elevated capillary density, decreased infarct size, and improved cardiac contractility in animal models [252]. Ultimately, these findings underscore the potential of exosome preconditioning to enhance the reparative efficacy of stem cell therapies by simultaneously modulating both the cellular phenotype and the surrounding microenvironment.

Collectively, these findings underscore the emerging paradigm of sequential or combinatorial exosome‐stem cell therapies in cardiac regeneration. Exosomes serve as powerful enhancers, not merely through paracrine support but by reprogramming stem cells and reshaping the ischaemic microenvironment to promote stem cell survival. Importantly, this synergy overcomes the major limitations of traditional stem cell therapies, offering a more robust and adaptable platform for myocardial repair. As such, future therapeutic protocols should consider integrating exosome preconditioning or co‐delivery into stem cell transplantation regimens, with precise attention to timing, source compatibility, and the molecular cargo of the exosomes used.

## 10 Conclusion

Exosome‐based therapies represent a promising evolution in regenerative medicine, particularly in myocardial repair, offering a cell‐free alternative that circumvents many of the limitations associated with direct stem cell transplantation [73]. However, the field currently lacks a standardised framework for the selection, dosing, delivery, and optimization of exosome therapies. This variability is further complicated by differences in source cell type, which critically shapes exosomal content, function, and clinical applicability. A comparative evaluation of CPC‐ex, MSC‐ex, and iPSC‐ex reveals distinct mechanistic advantages. CPC‐ex have demonstrated superior cardioprotective efficacy, particularly in promoting cardiomyocyte survival and angiogenesis, owing to their enriched cargo of anti‐apoptotic and pro‐angiogenic factors, as well as unique proteolytic mechanisms. These features make CPC‐ex highly suited for direct structural myocardial repair following acute infarction. In contrast, MSC‐ex display strong immunomodulatory and anti‐inflammatory profiles, making them ideal candidates for modulating the post‐infarction milieu and restoring cardiac function. Meanwhile, iPSC‐ex possess advantages related to delivering pluripotency‐associated miRNAs that modulate intracellular calcium cycling, oxidative stress, and vascular regeneration. Yet, complex reprogramming protocols limit their immediate translational utility. In practical application, this suggests a potential ranking of therapeutic utility: CPC‐ex as the most directly reparative in acute MI, MSC‐ex as optimal adjuncts for immune and inflammation modulation, and iPSC‐ex as a longer‐term strategy pending further refinement. Additionally, administration route, timing, preconditioning, and donor factors such as age and sex further influence therapeutic outcomes and require standardisation in future studies [80, 90]. Moving forward, comprehensive comparative studies are essential to refine dosing, optimise delivery strategies, and establish robust quality control for therapeutic exosomes. Crucially, the therapeutic efficacy of each exosome type must be matched to specific clinical contexts. Only through such systematic evaluation can exosome therapy reach its full potential in myocardial repair and beyond.

## Author Contributions

**Anthony Yazbeck:** formal analysis (lead), writing – original draft (lead). **Zena Wehbe:** formal analysis (lead), writing – original draft (lead). **Yara Menassa:** formal analysis (lead), writing – original draft (lead). **Assaad A. Eid:** writing – review and editing (equal). **Amirhossein Sahebkar:** writing – review and editing (equal). **Ali H. Eid:** conceptualization (lead), formal analysis (lead), project administration (lead), resources (lead), supervision (lead), writing – original draft (supporting), writing – review and editing (lead). **Alaa Abdelhamid:** writing – review and editing (equal).

## Conflicts of Interest

The authors declare no conflicts of interest.

## Data Availability

The authors have nothing to report.
